# Ceria-based nanotheranostic agent for rheumatoid arthritis

**DOI:** 10.7150/thno.49069

**Published:** 2020-10-25

**Authors:** Irina Kalashnikova, Seock-Jin Chung, Md Nafiujjaman, Meghan L. Hill, Mzingaye E. Siziba, Christopher H. Contag, Taeho Kim

**Affiliations:** 1 Department of Biomedical Engineering and the Institute for Quantitative Health Science & Engineering.; 2 Department of Microbiology and Molecular Genetics, Michigan State University, East Lansing, MI 48824, USA.

**Keywords:** theranostics, nanoceria, albumin nanoparticles, rheumatoid arthritis, ICG, imaging

## Abstract

Rheumatoid arthritis (RA) is an autoimmune disease that affects 1-2% of the human population worldwide, and effective therapies with targeted delivery for local immune suppression have not been described. We address this problem by developing a novel theranostic nanoparticle for RA and assessed its therapeutic and targeting effects under image-guidance.

**Methods:** Albumin-cerium oxide nanoparticles were synthesized by the biomineralization process and further conjugated with near-infrared, indocyanine green (ICG) dye. Enzymatic-like properties and reactive oxygen species (ROS) scavenging activities, as well as the ability to reprogram macrophages, were determined on a monocyte cell line in culture. The therapeutic effect and systemic targeting potential were evaluated in collagen-induced arthritis (CIA) mouse model using optical/optoacoustic tomographic imaging.

**Results:** Small nanotheranostics with narrow size distribution and high colloidal stability were fabricated and displayed high ROS scavenging and enzymatic-like activity, as well as advanced efficacy in a converting pro-inflammatory macrophage phenotype into anti-inflammatory phenotype. When administrated into affected animals, these nanoparticles accumulated in inflamed joints and revealed a therapeutic effect similar to the gold-standard therapy for RA, methotrexate.

**Conclusions:** The inflammation-targeting, inherent contrast and therapeutic activity of this new albumin-cerium oxide nanoparticle may make it a relevant agent for assessing severity in RA, and other inflammatory diseases, and controlling inflammation with image-guidance. The design of these nanotheranostics will enable potential clinical translation as systemic therapy for RA.

## Introduction

RA is a long-term, progressive disease characterized by chronic inflammation within the synovial tissues in multiple joints (synovitis), leading to increscent and erosive destruction of cartilage, joints, and the underlying bones 1; the importance of inflammation in RA is well understood 1-4. This chronic autoimmune disease is associated with elevated levels of reactive oxygen species (ROS), leading to increased lipid peroxidation, protein oxidation, and DNA damage 1. Elevated levels of ROS (superoxide anion, hydroxyl radicals, hydrogen peroxide, and singlet oxygen) generate oxidative stress and hypoxia that evoke the activation of several redox-sensitive transcription factors, including hypoxia-inducible factor-1 (HIF-1α). The factors that increase the expression of vascular endothelial growth factor (VEGF) responsible for intensive synovial angiogenesis and TLR/nuclear factor KappaB (NF-kB) 4. Activation of NF-kB results in the induction of pro-inflammatory gene expression and activation of a pro-inflammatory phenotype of macrophages (M1) 2, 5. Macrophages are one of the resident cell types in synovial tissue, along with synovial fibroblasts, T cells, and infiltrating monocytes 4. M1 macrophages produce effector molecules such as ROS, nitric monoxide, and inflammatory cytokines (interleukin (IL)-1β, tumor necrosis factor (TNF)-α), and chemokines, which regulate immune cell migration, differentiation, and activation. These factors are potentially toxic in instances of chronic inflammatory disease. Additionally, M1 macrophages secrete enzymes involved in driving an acute inflammatory response and joint destruction. IL-1β and TNF-α, the most abundant cytokines in the inflamed synovium, lead to synovial inflammation and further activate chondrocytes and synovial fibroblasts 2-5. Another type of macrophages, anti-inflammatory M2 macrophages, produces anti-inflammatory cytokines associated with tissue remodeling and immune-regulatory functions and, in the case of chronic inflammation, improve RA pathogenesis. The close link between ROS levels and the M1/M2 ratio in this disease has been established 2-5. Thereby the main goal of RA prevention and therapy should be to resolve inappropriate inflammation early to prevent joint damage and restore normal healthy tissues. A wide variety of anti-inflammatory therapies for RA have been developed. The RA drugs currently in use are combinations of non-steroidal anti-inflammatory drugs (NSAIDs), corticosteroids, and disease-modifying antirheumatic drugs (DMARDs). NSAIDs and corticosteroids have been shown to reduce patients' symptoms, and DMARDs, such as methotrexate (MTX), have long term effects that slow down disease progression 6-8. However, some RA patients do not respond to these drugs. Although the newer class of DMARDs can modulate cellular immune responses, they have many side effects 9. In this context, the unmet need in effectively treating and restoring immune homeostasis in RA is to develop novel agents that normalize elevated ROS levels, resolve hypoxia and restore the M1/M2 balance, which cumulatively would eliminate deleterious tissue damage due to chronic inflammation.

**Figure 1:**
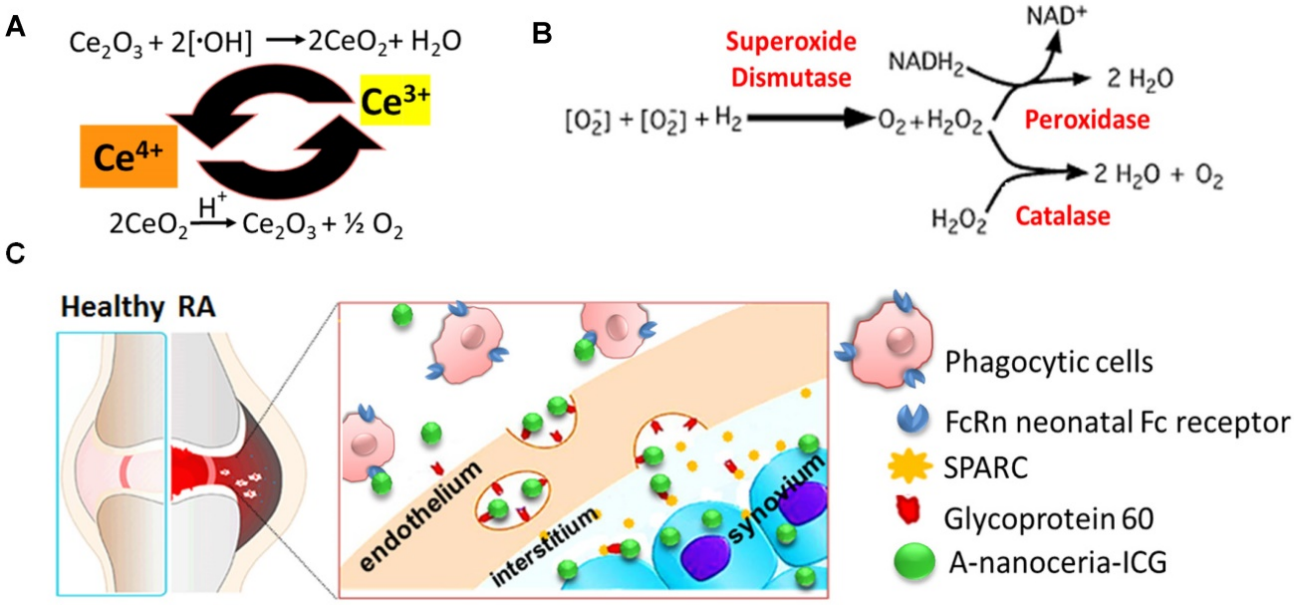
Schematic representation of **(A)** valence states recycling in nanoceria; **(B)** enzymatic modes of nanoceria; **(C)** albumin-binding receptors interaction with A- nanoceria within inflamed RA joint.

Various nanoparticles (NPs) have been described for RA treatment, including liposomes, polymeric nanoparticles, dendrimers, and metallic nanoparticles 9-11. Most NPs are designed as delivery vehicles for nucleic acid-based therapies or small molecules, and are used to target sites inflammation for treatment or imaging. Very few nanodrugs possess their own therapeutic attributes 11-15. Cerium oxide nanoparticles (nanoceria) are potent inhibitors of inflammation due to their ability to scavenge free radicals 12, 13, 16 (valence states recycling **Figure 1A** and enzymatic modes switch **Figure 1B**), and their ability to modulate downstream inflammatory targets (NF-kB, IL-6, and IL-8). They also increase transcription of antioxidant enzymes (superoxide dismutase, catalase) and affect oxidative stress markers (NADPH oxidase 2, glutathione peroxidase 4) 12, 13. Nanoceria has been shown to scavenge ROS generated in M1 macrophages and supply oxygen to successfully regulate hypoxia (HIF-1α), resulting in reduced aberrant angiogenesis and increased polarization of macrophages toward anti-inflammatory M2 subtypes 12, 13. Nanoceria has been shown to decrease M1 macrophage markers (TNFα, IL6, and VEGF) and to increase M2 macrophage markers (MMP 9, Platelet-derived growth factor) 17-20. Thus, the properties of nanoceria, over other nanomaterials used for RA therapy, appeared ideally suited as a therapeutic, but its delivery required some improvement.

In this study, we synthesized albumin-nanoceria NPs (A-nanoceria) *via* a biomineralization process 21 using hydrogen peroxide as an oxidizer. Interaction of hydrogen peroxide with both ceria and albumin resulted in the formation of nanozymes with unique antioxidant and enzymatic properties. Albumin is a natural protein and essential nutrient, known to exhibit extensive bioavailability with some apparent targeting to inflammation sites due to the local increased demand. Notably, several albumin and scavenging receptors are widely distributed in the inflamed joints in RA, and these are responsible for albumin uptake, transcytosis, and degradation 22-28. For example, gp60, glycoprotein 60, is overexpressed in the sera of patients with RA (RA mean concentration is 97 µg/mL relative to the normal mean concentration of 34 µg/mL), along with the Fc receptors being present at high levels in the phagocytic cells (macrophage, monocytes, dendritic cells) that are abundant at sites of inflammation 23. Another albumin-binding protein, the secreted protein acidic and rich in cysteine (SPARC), also known as a glycoprotein of the extracellular matrix that binds albumin 24, is overexpressed in RA synovial fluid and in murine collagen-induced arthritis (CIA) 25-27. Gp60 and SPARC work in conjunction to transport albumin across the endothelial barrier to the interstitium and then translocate the protein into immune cells and cells of the synovium. The possible targeting potential of A-nanoceria is presented with a schematic illustration (**Figure 1C**). The potential of A-nanoceria as a systemically delivered therapy for RA, i.e. *via* intravenous injection, seems high due to its long circulation time 27-30. Albumin is an endogenous protein, produced by the liver, and avoids capture by the reticuloendothelial system 27; on average, it has a 19-day half-life in human blood 27. Furthermore, albumin is chemically modifiable for the incorporation of fluorescent dye molecules that enable noninvasive visualization after accumulation at target sites. This has been shown in several previous diagnostic imaging studies with other albumin-indocyanine green (ICG) conjugates 29-31. The excitation/emission wavelength of ICG is in the near-infrared window (760/830 nm), which facilitates deeper tissue penetration than dyes with shorter excitation and emission wavelengths. ICG is already used clinically and is an FDA approved fluorophore that can be used in dual-modality imaging; fluorescence (optical) and photoacoustic (optoacoustic) 29-32. Interestingly, photoacoustic (optoacoustic) imaging, based on its 'light in/sound out' approach, can provide the information for *in vivo* tracking of blood clearance and accumulation of selected drug molecules or nanoparticles combined with the anatomical tomographic images from ultrasound 33-36.

In this study, we sought to demonstrate the theranostic potential of albumin-ceria-ICG formulations as an effective image-guided therapeutic for RA such that we could assess delivery and efficacy through imaging. We synthesized the multifunctional, theranostic nanoparticles (nanotheranostics) *via* biomineralization and conjugation chemistry, and further assessed its effects both on macrophage cell lines in culture and in a CIA mouse model for its potential to: 1) normalize disrupted ROS levels and resolve hypoxia, 2) restore a balance of pro-inflammatory (M1) and increase anti-inflammatory (M2) macrophages *via* reprogramming M1-like to M2-like macrophage phenotypes; and 3) provide real-time monitoring of therapeutic delivery, distribution, and half-life circulation by optical/optoacoustic imaging. In this way, we describe a new type of anti-inflammatory nanodrug delivered systemically to image and treat RA.

## Methods

### Materials

Cerium nitrate hexahydrate (99.999% purity; catal. #202991), hydrogen peroxide (catal. #216763), bovine serum albumin (BSA, catal. #05470), methotrexate hydrate (catal. #A6770), dimethyl sulfoxide (DMSO, catal. #D2650), boric acid (catal. #B0394), sodium hydroxide (catal. #S8045), phorbol 12-myristate 13-acetate (PMA, catal. #P1585), ammonium hydroxide (catal. #221228), dialysis tubing cellulose membrane with 14kDa cut off (catal. #D9277-100FT), sodium azide (catal. #S2002), peroxidase assay kit (catal. #MAK092), anti-β-Actin antibody (catal. #A1978), normal goat serum (catal. #9023), and lipopolysaccharide (LPS, catal. #L2630) were purchased from Sigma-Aldrich Chemicals (Atlanta, GA, USA). Trypsin/EDTA (catal. #25200056), dulbecco's modified eagle's medium (DMEM, catal. #11965092), phosphate buffered saline (PBS, catal. #10010023), fetal bovine serum (FBS, catal. #16000044), 3-(4,5-dimethylthiazol-2-yl)-2,5-diphenyl tetrazolium bromide) (MTT, catal. #M6494), penicillin/streptomycin (catal. #15070063), BCA assay (catal. #23227), Arc Reactive Beads (catal. #A10628), 4% paraformaldehyde in PBS (catal. #AAJ19943K2), 20 kDa cut off mini-dialysis Slide-A-Lyzer units (catal. #69570), 2,7-dichlorodihydrofluorescein diacetate (DCFDA, catal. #C6827), Bel-Art™ SP Scienceware™ Flowmi™ cell strainers for 1000 µL pipette tips (catal. #14-100-150), Anti-CD11b antibody (catal. #PA5-79532), TRIzol™ Reagent (catal. #15596026), Pierce Protease Inhibitor Mini Tablets (catal. #PIA32955) and Pierce™ ECL Western Blotting Substrate (catal. #32209) were purchased from Thermos Fisher (Waltham, MA). Human and mouse TruStain FcX (catal. #422302& 101319), Zombie NIR dye (catal. #423105), PE-CY5 CD80 (catal. #305209), APC/Fire-750 CD80 (catal. #104739), AF647 CD209 (catal. #330111), BV421 CD206 (catal. #141717) were purchased from BioLegend (San Diego, CA). Anti-mouse and anti-rat/hamster BD comp beads (catal. #552845 and 552843) were purchase from BD Biosciences (San Jose, CA). Amicon ultra-15 centrifugal filter Units with 100 kDa cut off were purchased from EMD Millipore (Burlington, MA). IL*-*4 (catal. #780451&78047), IL*-*13 (catal. #78030 & 78029), IFN*γ* (catal. #78020) were purchased from STEM CELLS Technologies (Cambridge, MA). ICG-NHS ester (catal. #BCT-POS1604-M005) was purchased from Adipogen Life Sciences (San Diego, CA). Superoxide dismutase (SOD) and catalase assay kits were purchased from Cayman chemicals (Ann Arbor, MI). All chemicals were of analytical grade and used without further purification. All aqueous solutions were prepared with deionized (18 MΩ) water (DI water). Freund's adjuvants (complete: catal. #7001, incomplete: catal. #7002) and type II collagen (catal. #20022) were purchased from Chondrex Inc (Redmond, WA). SsoAdvanced™ Universal SYBR® Green Supermix (catal. #1725270) and 4-20% Mini-PROTEAN® TGX Stain-Free™ Protein Gels (catal. #4568093) were purchased from Bio-Rad (Hercules, CA). Anti-Arginase-1 (Arg-1) antibody (catal. #93668), Anti-HIF-1α antibody (catal. #36169), anti-rabbit IgG HRP-linked Antibody (catal. #7074), and anti-mouse IgG HRP-linked Antibody (catal. #7076) were purchased from Cell Signaling Technology (Danvers, MA). PrimeScript reverse transcription (RT) Master Mix was purchased from Takara (Otsu, Japan). Anti-iNOS antibody (catal. #sc-651) was purchased from Santa Cruz Biotechnology (Santa Cruz, CA).

### Synthesis of albumin-cerium oxide nanoparticles (A-nanoceria)

Bovine serum albumin (BSA) in DI water at 5 mg/mL concentration was mixed with 10 mg of cerium nitrate hexahydrate (99.999% purity) for 30 min at 37 °C with a pH of 8.0. Then 300 µl of 30% of hydrogen peroxide was added, and the solution was left stirring for an additional 2 h at 37°C. The sample was washed three times with DI water using 100 kilodalton (kDa) centrifuge filters at 3240 revolutions per min (rpm) for 10 min at 4 °C and dialyzed overnight in a cold room against DI water using 14 kDa cut off bags. Concentration of cerium oxide was determined through inductively coupled plasma-optical emission spectroscopy (ICP-OES, PerkinElmer) at 407.57 nm and using a calibration curve within 10-0.01 part per million (ppm) range. Concentration of BSA was determined with a BCA assay using a plate reader (VICTOR Nivo, PerkinElmer) and applying calibration curve for the range 0.05-1.5 mg/mL at 562 nm.

### A-nanoceria conjugation with indocyanine green (ICG)

Nanoparticles suspension was transferred into borate buffer with pH 7.9 where a small aliquot of ICG-NHS ester reconstituted in DMSO was added to the NPs. Molar ratio albumin to ICG of 1:15 was applied for this reaction that occurred at room temperature (RT for 4h. After that NPs were purified by dialysis against DI water using 20kDa cut off mini-dialysis Slide-A-Lyzer units for 48h in a cold room. Optical density (OD) of prepared NPs was determined at 780 nm by plate reader (Viktor Nivo, PerkinElmer) and ICG concentration was calculated using extinction coefficient 2.621 × 10^5^ M^-1^cm^-1^.

### Nanoparticles characterization

Hydrodynamic size and surface zeta potential measurements were determined using a dynamic light scattering (DLS) instrument (Zeta Sizer Nano, Malvern Instruments). Small aliquots of stock solution of each sample were diluted with DI water. For fourier-transform infrared spectroscopy (FT-IR) analysis, stock suspension of NPs were freeze-dried for 72 h, and the obtained powder was analyzed by FT-IR spectrometer equipped with an attenuated total reflection (ATR) element (PerkinElmer Spectrum One), operated in the range 4000-550 cm^-1^ with accumulating 20 scans. Full spectra of each sample were recorded by NanoDrop™ 2000/2000c Spectrophotometers (Thermo-Fisher) in the range 200-820 nm. The A-nanoceria surface chemistry of the top 50-80 angstroms (Å) was determined with X-Ray Photoelectron Spectroscopy (XPS). The measurements were performed using a PHI 5400 ESCA system. The base pressure of the instrument was less than 10^-8^ Torr. A 1 cm^2^ freeze-dried sample was mounted onto the sample holder with double-sided copper tape. The X-Ray was a non-monochromatic Mg source with a take-off angle of 45 degrees. Two types of scans were performed for each sample; a survey scan from 0-1100 eV taken with a pass energy of 187.85 eV and a regional scan of each element at a pass energy of 29.35 eV. Data was fit using PHI Multipak (v8.0) software. Transmission electron microscope (TEM) and scanning electron microscope (SEM) images were obtained by using JEOL 2200FS ultra-high-resolution TEM and JEOL 7500F SEM, respectively.

### Cell culture

Two cell lines, human monocytes THP-1 (ATCC TIB-202) and murine macrophage RAW 264.7 (ATCC TIB-71), were used. Cells were maintained in DMEM supplemented with 10% FBS and 1% penicillin/streptomycin. These adherent cell lines were cultured in humidified atmosphere containing 5% CO_2_ at 37 °C and removed from the plastic substrate using a 0.25% trypsin/EDTA solution for passage and prior to evaluation.

### Cell viability test

The RAW 264.7 cells or THP-1 (3000 cells/well) were seeded (N (wells) = 10) in 96-well plates for 24 h. Then cells were incubated with 100 µl of A-nanoceria at different concentrations reconstituted in cell culture medium without phenol red for 24 h at 37 °C. After, the cells were rinsed twice with PBS and then 100 µl of 5 mg/mL MTT reagent in cell culture medium without phenol red was added to each well of the plate and plate was incubated for 3.5 h at 37 °C. The assay was completed by aspirating the MTT reagent and adding 100 µl of DMSO. A plate was placed on orbital shaker for 15 min at RT to help the formazan crystals dissolve. Optical density (OD) in each well was determined at 550 nm by plate reader.

### Cell uptake study

Cells were seeded in 6 well plate at a density of 3×10^5^ cells per well and left to adhere overnight. The cells were then treated for 24 h with 50, 5, and 0.5 μg NPs per mL of cell culture medium. At the end of treatment, the cells were washed 3 times with PBS, and 300 μL of 0.5% trypsin/EDTA was added remove cells from the plastic substrate. Cells were collected by adding 500 µL of PBS. Cells pellets were treated with nitric acid (70%) for 72 h. Later, the samples were filtered using 0.2 μm syringe filters and diluted with DI water up to 2% of nitric acid in the sample. The amount of nanoparticles taken up was determined by ICP-OES.

### Intracellular reactive oxygen species (ROS) assay

The ROS assay is based on the conversion of oxidative sensitive dye, 2,7-dichlorodihydrofluorescein diacetate (DCFDA), into highly fluorescent 2,7-dichlorofluorescein (DCF) upon interactions with intracellular ROS. Cells seeded in 96-well plates (N (wells) = 10) were treated with different concentrations of A-nanoceria (0.5-50 µg/mL) and controls (untreated and 10 µg/mL LPS treated cells) for 24 h. At the end of treatment, the cells were washed three times with PBS, and exposed to 100 μL of 50 μM DCFDA in PBS for 30 min, protected from light. DCFDA was removed and the cells were washed with PBS again. Fluorescence was measured at 485 nm excitation wavelength and 520 nm emission wavelength by a plate reader (n = 5). Fluorescence intensities were normalized to the live cells based on MTT data.

### Enzymatic assays

SOD, catalase, and peroxidase assays were performed based on manufacturers' protocols (Cayman Chemicals and Sigma-Aldrich) using RAW 267.4 cells and A-nanoceria (50 µg/mL, N (wells) = 5). SOD activity was assessed by measuring OD at 440-460 nm with the dismutation of superoxide radicals generated from tetrazolium salt to formazan by xanthine oxidase and hypoxanthine. Catalase activity was measured with the reaction of methanol conversion into formaldehyde at optimal concentration of H_2_O_2_ where produced formaldehyde was determined colorimetrically with 4-amino-3-hydrazino-1,2,4-triazole (Purpald) at 540 nm. Peroxidase catalyzes the reaction between H_2_O_2_ and the probe, resulting in a colorimetric (570 nm)/fluorometric (λex = 535 nm / λem = 587 nm) product, proportional to the peroxidase activity present.

### Flow cytometry

RAW 264.7 cells were seeded (300,000 cells/mL) in 6-well plates, incubated overnight, and then treated with NPs at 0.5 ug/mL concentration in cell culture medium without phenol red for 24 h. Following, culture medium was aspirated; cells were rinsed twice with PBS, and detached by using 0.25% trypsin/EDTA solution. THP-1 cells were treated similarly, but before exposing them to NPs, they were treated with PMA 20 ng/mL for 24 h, then washed and rested for 24 h. The positive controls for M1 and M2 macrophage were prepared by cells treatment either with 40 ng/mL of IL-4/IL-13 or combination 10 ng/mL LPS with 20 ng/mL IFNγ for 24 h. After treatment cell pellets were re-suspended in flow wash buffer (1% BSA + PBS + 0.1% sodium azide) and incubated with anti-mouse or anti-human TruStain FcX for 10 min at RT, and then cells were stained with anti-mouse or anti-human antibodies (BV421 CD206, AF647 CD209 and APC/Fire750 CD80, Pe-Cy5CD80). After washing with PBS, cells were stained on ice with Zombie NIR at 1:500 dilution for 20 min. After staining, cells were washed twice with 1 mL of flow wash buffer (1% BSA + PBS + 0.1% sodium azide) and fixed with 500 µL of 1% paraformaldehyde in PBS for 30 min on ice. After that, 1 mL of flow wash buffer was added to each tube, and cells were centrifuged at 1000 rpm for 5 min at 4 °C. Before analysis, cell pellets were re-suspended in 300 μL of flow wash buffer and filtrated using 70 µm flowmi tip strainer. We performed flow cytometry study using Cytek Aurora (Cytek Biosciences) with FlowJo software (v10.6.2). Three separate flow cytometry studies were done.

### qRT-PCR analysis

Total RNA was extracted from cells using TRIzol reagent following the manufacturer's instructions. 1 µg of RNA was converted into complementary DNA using a complementary DNA synthesis kit. The expressions of IL-1β and iNOS were quantified using SYBR Green real-time PCR Master Mix with 2 ng of complementary DNA and 0.2 mM primers. Expression of the housekeeping gene β-actin was used to standardize IL-1β and iNOS expression. IL-1β primer 5'-GCAACTGTTCCTGAACTCAACT-3' (sense), 5'-ATCTTTTGGGGTCCGTCCAACT-3' (antisense), iNOS primer 5'-TTGGAGCGAGTTGTGGATTTG-3' (sense), 5'-GTAGGTGAGGGCTTGGCTGA-3' (antisense), and β-actin primer 5'-CGTGCGTGACATCAAAGAGAA-3' (sense), 5'-TGGATGCCACAGGATTCCAT-3' (antisense) were used. Quantitative RT-PCR (qRT-PCR) was performed using an Applied Biosystems 7500 (ThermoFisher, Waltham, MA) with the following parameters: 50 °C for 2 min, 95 °C for 10 min, and 40 cycles of 95 °C for 15 s and 60 °C for 1 min.

### Western blotting

Total protein was isolated from the cells using radioimmunoprecipitation assay buffer and protease inhibitor. 20 µg of proteins were separated in 8-20% gradient polyacrylamide gels and transferred onto PVDF membranes. The membranes were blocked with 5% skim milk for 30 min at room temperature and incubated overnight at 4 °C with primary antibodies (HIF-1α and β-actin). After that, membranes were probed with horseradish peroxidase-conjugated anti-rabbit or anti-mouse IgG. Immunoreactive bands were visualized with chemiluminescence reagents and captured using a ChemiDoc^TM^ (Bio-Rad, Hercules, CA) imaging system.

### Animal study: CIA model preparation and clinical score evaluation

All experimental procedures were approved by the Institutional Animal Care and Use Committee (IACUC) of Michigan State University. All genetically susceptible mice (DBA/1) mice were purchased from The Jackson Laboratory (Bar Harbor, ME). Arthritis was induced in collagen-induced arthritis (CIA) model by immunization with an emulsion of Freund's adjuvant and type II collagen, intra-dermally. In murine CIA, DBA/1J mice are immunized with a type II bovine collagen emulsion in complete Freund's adjuvant (CFA), and receive a boost of type II bovine collagen in incomplete Freund's adjuvant (IFA) 21 days after the first injection. After second injection for CIA modeling (day 21), mice were randomly divided into groups (N = 4-5 mice per group) and each group was given an injection of 1 mg/kg nanoceria (intra-articular injection, A-nanoceria), 1 mg/kg MTX (intraperitoneal (IP) injection), a widely used RA drug, or saline (intra-articular injection), and 1 mg/kg bovine serum albumin (intra-articular injection) two times a week. Disease severity in CIA is commonly assessed by clinical scoring of paw swelling. Clinical score was graded as follows which mentioned other studies: 0 = normal; 0.5 = erythema and edema in only one digit; 1 = erythema and mild edema of the footpad, or ankle or two to five digits; 2 = erythema and moderate edema of two joints (footpad, ankle, two to five digits); 3 = erythema and severe edema of the entire paw; 4 = reduced swelling and deformation leading to incapacitated limb. After the second injection, the severity of the arthritis was scored three times a week. *In vivo* imaging experiments were performed in mice (N = 3 per each group) at three weeks after the second injection.

### IVIS study and analysis

Fluorescence images were obtained using an IVIS *in vivo* imaging system (PerkinElmer, Waltham, MA) for data acquisition and analysis. PBS, ICG and ICG-conjugated nanoparticles (50 µg and 30 µM ICG) were intravenously injected in mice, and images were taken after 0, 0.5, 1, 2, 4, 8, 16 and 24 h. Mice were kept under anesthesia with 2% isoflurane, and fluorescence imaging was performed with excitation 745-nm and emission 820-nm (binning 8, exposure 5 s). SPARC-targeting mechanism of A-nanoceria uptake was evaluated by IVIS imaging in CIA mice (N = 3 mice). 50 µg of mouse anti-SPARC antibody (ab55847, Abcam) were IP injected 1 h earlier, after then ICG-conjugated A-nanoceria with A-nanoceria precursor (1:3 ratio) were tail intravenously injected. All data are shown as means (5 hind paws per each group) ± SEMs. Statistical significances were determined using the Student's t-test or Mann-Whitney U test, and *P*-values of < 0.05 were considered statistically significant.

### MSOT study and analysis

All photoacoustic images were obtained using a multispectral optoacoustic tomography (MSOT) system (iThera Medical, Munich, Germany). Mice (N = 2, 4 paws) are submerged in a water tank in a horizontal position in a holder and are wrapped in a thin polyethylene membrane to prohibit direct contact between water and mouse but still allow for acoustic coupling. Anesthesia and oxygen are supplied through a breathing mask. The images were taken 0 and 24 h after intravenously injection of ICG-conjugated nanoparticles (100 µg/50μL and 30 µM ICG) in mice. Imaging range was from the toes to the hips using 0.5 mm steps, and all acquisition was performed using 10 averages per illumination wavelength, with the wavelengths chosen as follows: 680, 700, 730, 760, 800, and 850 nm. This resulted in an acquisition time of less than 20 min. Image analysis was performed by using ViewMSOT software suite supplied with the iThera Medical system.

### Immunohistology

After 3 weeks of treatment, joints (N = 2) of normal, PBS or A-nanoceria treated mice were amputated and decalcified for one week at 4 °C in a solution with 14% ethylenediaminetetraacetic acid (pH 7.2). After decalcification, tissues were fixed in 10% paraformaldehyde for one day at room temperature, and prepared for frozen embedding. 5 μm of sagittal sections from the center of joint were used for slides. For staining, sections were permeabilized in 0.5% Triton X-100 for 5 min. The sections were blocked with goat serum (diluted 1:30 in PBS) for 1hr at room temperature and incubated overnight with primary antibodies for iNOS, Arg-1, and CD11b at 4 °C. After washing with PBS, Alexa 555-conjugated secondary antibodies were incubated 1hr at room temperature. The slides were mounted using a mounting reagent with DAPI. Fluorescence images were obtained using an epifluorescence microscopy (ECLIPSE Ts2R, Nikon Instruments).

### DATA quantification and statistical analysis

A region of interest (ROI) was drawn around the hind paw of mice obtained from IVIS and MSOT images and the background signal of the ROI was removed. Mean values, standard deviations, and *p*-values were calculated in Microsoft Excel. All error bars represent the standard deviations except the clinical scoring evaluation study and imaging study, where we used the standard error of the mean (SEM). A two-tailed Student's t tests as well as Mann-Whitney U test was used to determine statistical significance and p-values of < 0.05 were considered to be significant. Measurements and experiments were performed in triplicate unless otherwise specified.

## Results and Discussion

### Nanotheranostics preparation and characterization

**Figure 2:**
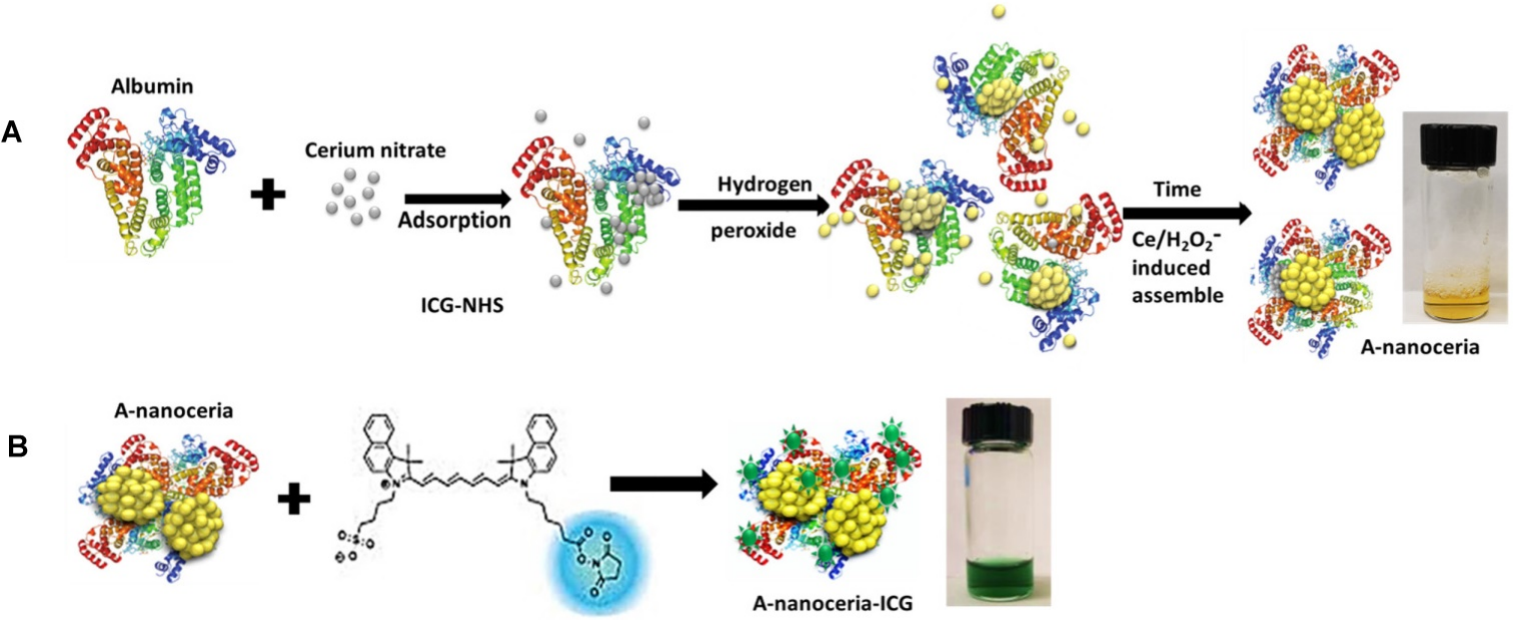
Schematic representation of **(A)** albumin-nanoceria formation (inset: a picture of transparent, nanoparticle suspension with A-nanoceria) and **(B)** A-nanoceria-ICG synthesis (inset: a picture of nanoparticle suspension with A-nanoceria-ICG).

**Figure 3:**
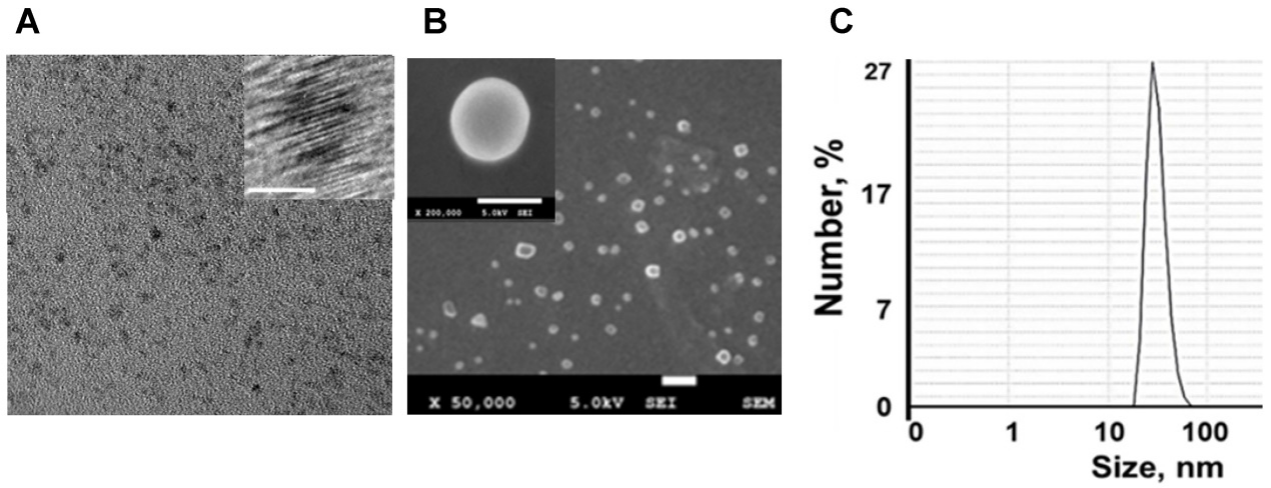
** A-nanoceria characterization by zetasizer and electron microscopies. (A)** TEM (20 nm bar) and HR-TEM (inset, 5 nm bar) images; **(B)** SEM (100 nm bar) and HR-SEM (inset, 50 nm bar) images; **(C)** Hydrodynamic diameter of A-nanoceria.

**Figure 4:**
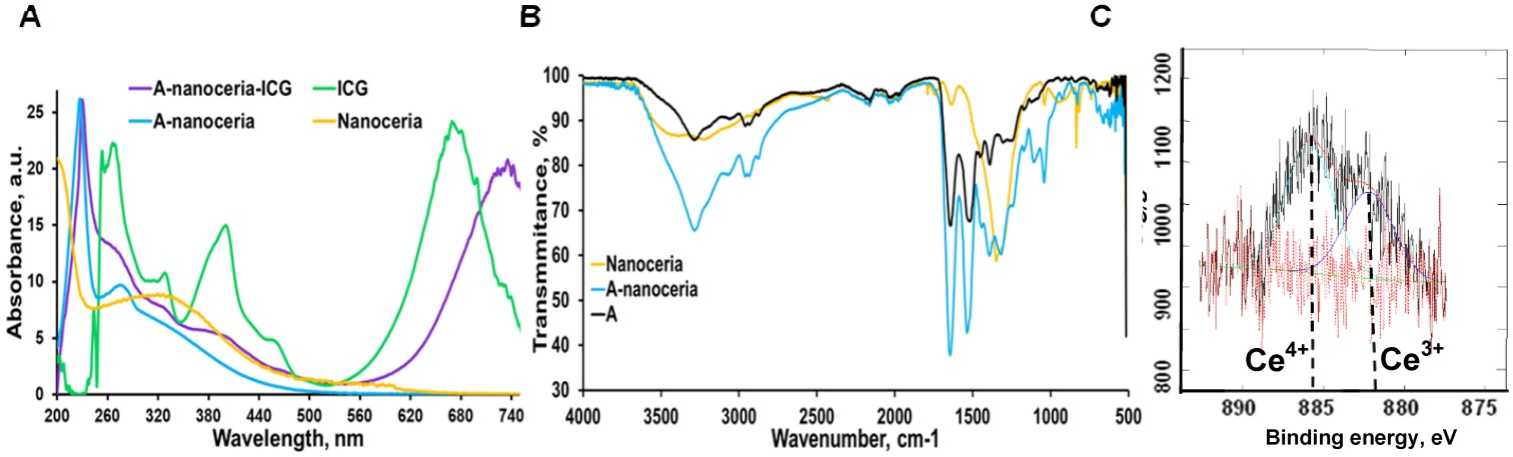
A-nanoceria characterization by **(A)** UV-Vis (yellow: nanoceria, blue: A-nanoceria, green: ICG, purple: A-nanoceria-ICG); **(B)** FT-IR (yellow: nanoceria, blue: A-nanoceria, black: albumin); **(C)** XPS results, confirming the mixed valence states of Ce^3+^ and Ce^4+^ (59.37% of Ce^3+^ on 882.25 eV, 40.63% of Ce^4+^ on 885.9 eV). Full band description for **(A)** and **(B)** can be found in Supplementary Materials (**Table S1**). Full XPS spectra for **(C)** can be found in **Figure S2C**.

**Figure 5:**
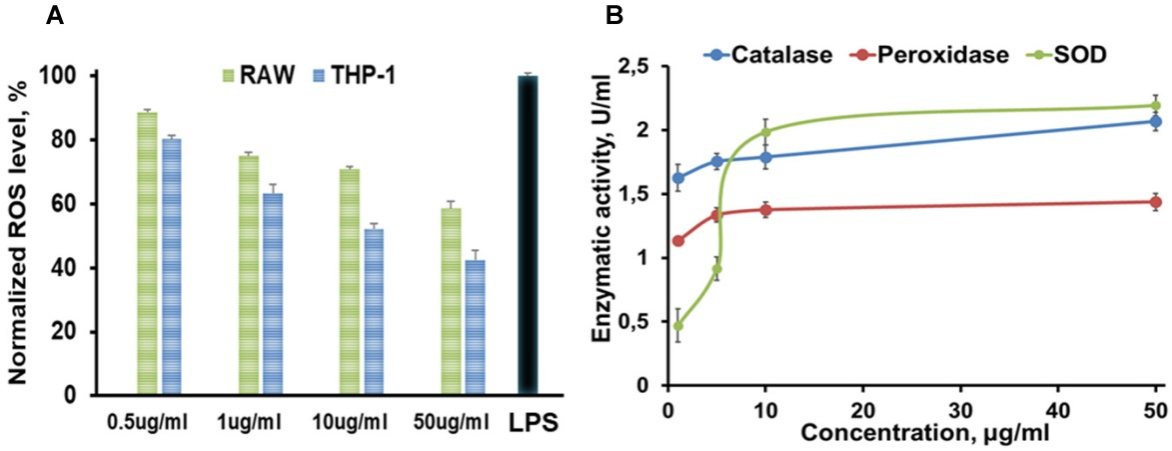
Summarized *in vitro* results of **(A)** ROS assay (green: RAW 267.4 and blue: THP-1 cells were treated with 0.5, 1, 10, 50 µg/mL A-nanoceria , and with LPS as a control), where N = 10, error bars = SD; 2.4 and 1.7-fold decrease on ROS in THP-1 and RAW cell, respectively, were detected after treatment with 50 µg/mL A-nanoceria); **(B)** Enzymatic-like activity of A-nanoceria tested on RAW 267.4 cells at 0.5, 5, 10, and 50 µg/mL A-nanoceria, where N = 5, error bars = SD. The green line: SOD, blue line: catalase, red line: peroxidase. Enzymatic-like activities of A-nanoceria is concentration dependent. SOD and catalase-like activities of A-nanoceria at 50 µg/mL are close.

Nanotheranostics comprised of bovine serum albumin (A) and indocyanine green (ICG) dye were developed to deliver cerium oxide nanoparticles effectively in a trackable manner to inflamed joints for RA therapy. A general mechanism of protein-metal nanoparticle formation has been proposed in 37 and consists of three different steps, (i) the ionic binding of metal (Me) ions with different amino acid residues of the protein, (ii) nucleation, reduction of Me^n+^ to Me(0), and (iii) coalescence, the growth of Me nanoparticles 37. Likewise, cerium oxide nanoparticles could be growing on albumin substrate *via* the biomineralization process but with the assistance of hydrogen peroxide as an oxidizer. Under oxidative stress by hydrogen peroxide, Cys34 thiol of albumin shifts to an exposed conformation and is oxidized into sulfenic acid (A-Cys-SOH) 38. Further, A-Cys-SOH could coordinate ceria ions, forming a new A-ceria structure similar to one observed in the enzymes 39. Thereafter, small intermediate complexes of albumin-ceria could assemble into larger structures (**Figure 2A**) *via* sulfenic acid conversion into intramolecular disulfide (A-S-S-A) 40. Convenient synthesis without the utilization of any harsh conditions (high pH and temperature, a denaturating agent) as previously reported 21 led to the formation of uniform, water-soluble albumin-ceria nanoparticles (A-nanoceria) with unique biological identity, high reproducibility, and easy scale-up. High colloidal stability of as-prepared NPs is another benefit of the protein-mediated approach. It was confirmed that A-nanoceria nanoparticle suspension has much better aqueous stability than bare nanoceria synthesized *via* the same chemistry but without substrate attendance (**Figure S1**). Further, A-nanoceria was conjugated with ICG *via* amino groups-NHS-chemistry to make the nanoparticles more visible through optical imaging (**Figure 2B**). The synthesized A-nanoceria-ICG present small sizes with CeO_2_ core ≈ 5 nm (4.9±1.1 nm by ImageJ) from TEM images (**Figure 3A**), and together with protein shell 30 nm (**Figure 3B**) (30±8.9 nm from SEM images treated by ImageJ), and 98±1.3 nm (PDI: 0.34±0.04) from DLS data (**Figure 3C**). CeO_2_ core has a highly crystalline structure detected from HR-TEM (**Figure 3A inset**). Zeta potential of A-nanoceria was pH-dependent (**Figure S2A**). The NPs stayed positively charged up to their isoelectric point (Ip) at pH 5.4 and became negatively charged within pH 5.5-7.4 (**Figure S2A**). So, the NPs should carry negative change into the inflamed synovium displayed pH around 6.0-7.0 41. The negative charge of NPs usually reduces the undesirable clearance by the reticuloendothelial system and improves circulation time 42; this effect may even be doubled in our approach due to the albumin-based shell of the NPs. The presence of cerium oxide (CeO_2_) within NPs was confirmed by optical spectroscopy analysis (**Figure 4A**), ICP-OES analysis, and EDS from SEM analysis (**Figure S2B**). Typical UV-Vis peak in the spectrum of nanoceria (**Figure 4A**) (230-260 nm range absorbance) corresponding to cerium (III) was shifted due to the albumin substrate. The second peak absorbance occurring in the 300 to 400 nm range and corresponding to cerium (IV) absorbance 43 presents in A-nanoceria with some shift as well (**Figure 4A**). Successful ICG conjugation was validated with the absorption band in the 670-730 nm (**Figure 4A**). FT-IR spectroscopy results have also corroborated the existence of both cerium oxide and albumin (**Figure 4B**). The summarized spectroscopy data are gathered in **Table S1**. The X-ray photoemission spectroscopy (XPS) revealed a different ratio of Ce (III) to Ce (IV) ions (**Figure 4C and Figure S2C**). Most nanoceria characteristics are endowed by the co-existence of both Ce^4+^ and Ce^3+^ oxidation states and their recycling *via* oxidation/reduction reactions (**Figure 1A**). A-nanoceria contains Ce (III) ions slightly more prevailing over Ce (IV) ions (**Figure 4C**). The ability to recycle between two valence states (Ce^3+^ and Ce^4+^) within nanoceria structure as well as Ce^3+^/Ce^4+^ ratio is determinative in the success of ROS scavenging and reprogramming of macrophage phenotype after being uptaken by immune cells 12, 13, 17, 18, 44. Fabricated A-nanoceria exhibits excellent ROS scavenging capacity decreasing ROS released by macrophages and monocytes induced by LPS (**Figure 5A**). The cytotoxic effect of NPs may depend on their uptake, whereas the latter may define NPs' therapeutic effect. Both parameters, cytotoxicity and uptake of A-nanoceria, were evaluated *in vitro* using immune cells, and the results are presented in **Figure S2D-E**.

### Enzymatic features of A-nanoceria

The etiology of RA is multifactorial and involves ROS, oxidative stress, and imbalance between oxidants and antioxidants in the pathogenesis of the disease, and harmful effects of M1 macrophages being out of balance with M2 macrophages 2-5. Abundant oxidative stress in RA patients as evidenced by increased intracellular ROS production, increased lipid peroxidation, protein oxidation, DNA damage, and impaired enzymatic/non-enzymatic antioxidant defense system of the body has been verified 1, 4. There is a growing awareness that ROS and free radicals may play an essential role in mediating cellular injury and tissue damage in RA 1-4, 45-47. Antioxidant therapy, including non-enzymatic and enzymatic (SOD, catalase, glutathione peroxidase, glutathione reductase, and glutathione-S-transferase), may lead to protection against inflammatory diseases including RA 48-50. For example, SOD, a cytoplasmic enzyme related to the removal of superoxide radicals, may contribute to the pathogenesis of RA, and has long been used for antioxidant therapy 49, 50. SOD can also mediate a reduction of inflammation by inhibiting macrophage migration into injured tissues. However, the application of SOD in RA therapy is limited due to its short half-life in blood, rapid renal clearance, and especially low accumulation at targeted sites 48-50. Besides that, SOD is the only first line of defense against the free radical formation and is required to dismutation superoxide radical into hydrogen peroxide (**Figure 1B**). Further catalase and glutathione peroxidase are necessary to take over the antioxidant role catalyzing the conversion of hydrogen peroxide into water and oxygen (**Figure 1B**) and hence protects the cells from the pernicious effects of accumulated hydrogen peroxide. Notably, nanoceria operates as a multiple-enzyme to mimic the biological actions of SOD, catalase, and peroxidase (**Figure 1B**), as well as it can scavenge ROS directly *via* Fenton reaction 12, 13. Enzymatic activity of nanoceria mostly arises from quick and expedient switches in oxidation states between Ce^4+^(CeO_2_) and Ce^3+^(Ce_2_O_3_) (**Figure 1A**) for scavenging of ROS 13, 17, 18, 45, 46. The enzymatic activity of A-nanoceria was evaluated as an overall ROS scavenger capacity at different concentrations of the NPs (**Figure 5B**). A-nanoceria possesses the activity of SOD and catalase/peroxidase (**Figure 5B**) due to the coexistence of Ce^3+^/Ce^4+^ in the structure of A-nanoceria (**Figure 4C**). Thus, nanoceria may contribute to normalizing oxidant/antioxidant levels, acting as these three enzymes.

### Macrophage polarization *in vitro* and* in vivo*

**Figure 6:**
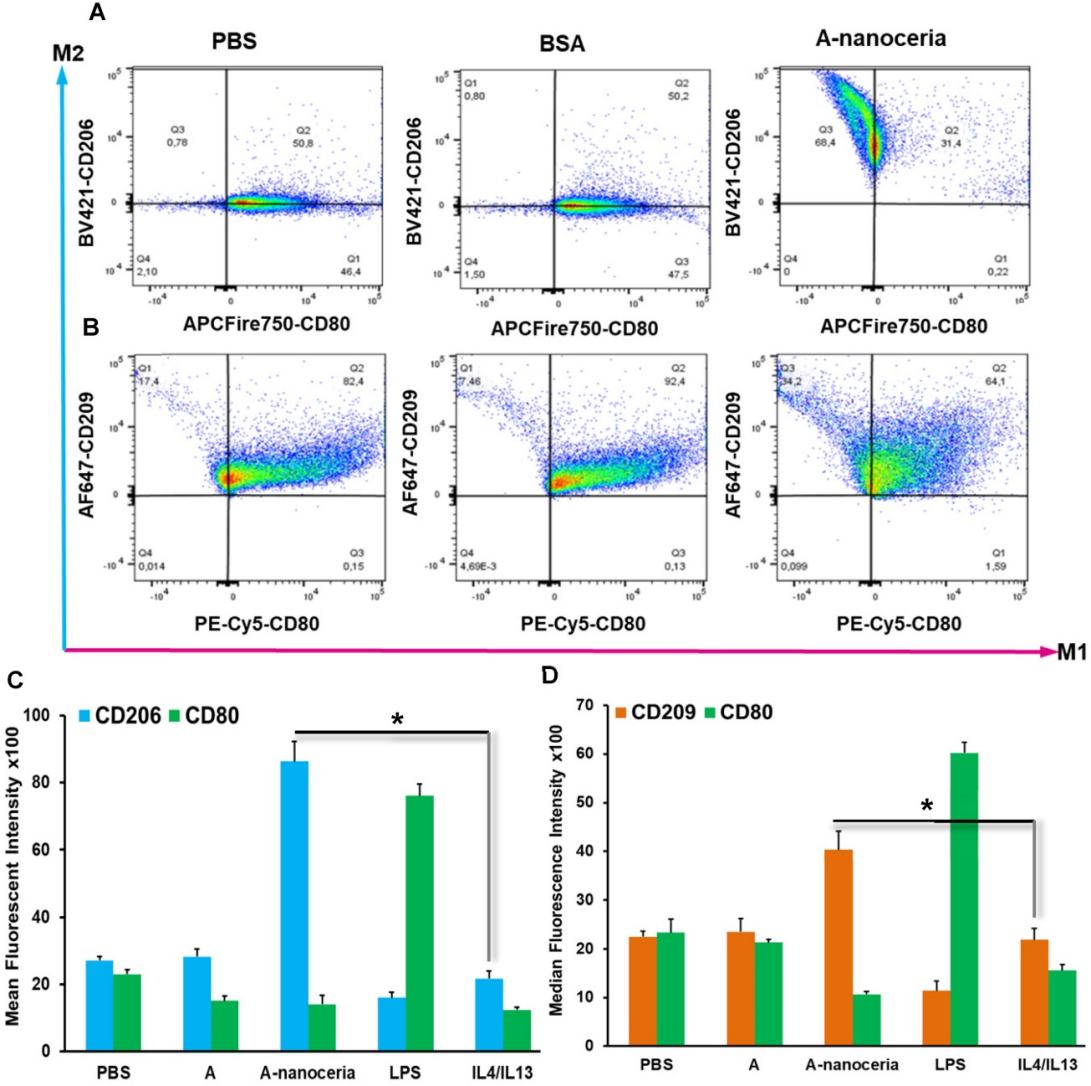
Flow cytometry analysis of **(A)** RAW 264.7 cells and **(B)** THP-1 cells. A-nanoceria treated cells displayed clear shift to M2-like phenotype (Q3 68.4% and 34.2%) compared control samples (PBS and BSA); Mean fluorescent intensity (MFI) data for **(C)** RAW 264.7 cells and **(D)** THP-1 cells. Samples treated with A-nanoceria (CD206^high^ or CD209^high^ and CD80^low^) showed much intensive response than samples treated with IL4/IL13 (N = 3, error bars = SD, p-value: *p < 0.05).

**Figure 7:**
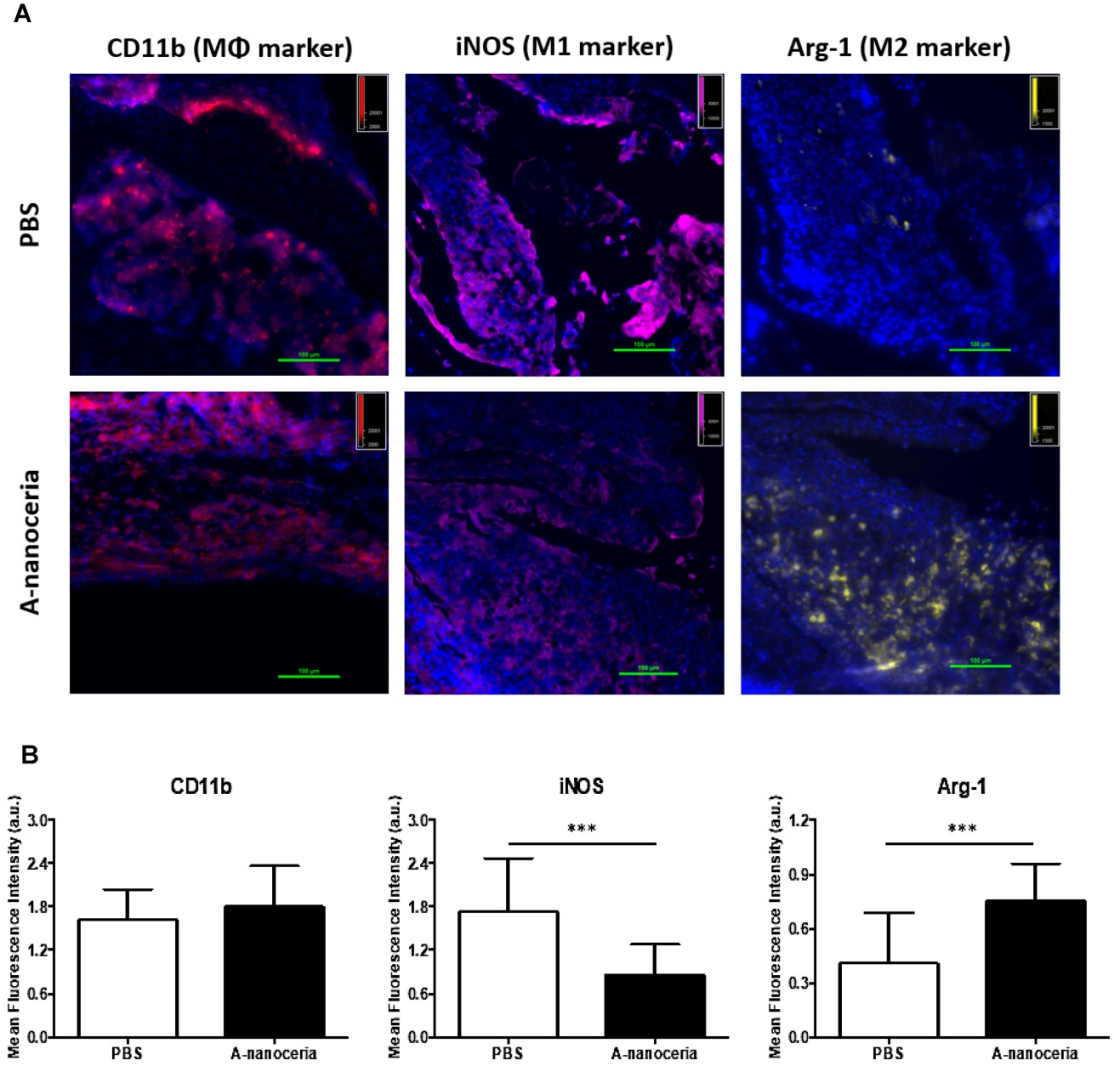
** Macrophage polarization in tissue sections harvested from PBS and A-nanoceria treated groups of CIA mice by immunohistology. (A)** Images of tissue sections from animals treated with PBS or A-nanoceria. Sections of inflamed tissues (DAPI in blue) displayed a high concentration of infiltrating macrophages into the paws (CD11b in red). The number of M1 macrophages (iNOS in pink) decreased upon treatment with A-nanoceria. Tissues from animals treated with A-nanoceria revealed a high number of M2 macrophages (Arg-1 in yellow). Immunohistology of tissue sections harvested from normal mice can be found in **Figure S6**;** (B)** Summarized plot of CD11b, iNOS and Arg-1 MFI signals in ROI from images. In each group, we took 5 fluorescence images from two mice tissues and plotted 5 random ROIs for each image (N = 25. error bar = SD, *p*-value: ***p < 0.0005).

Macrophages are the most abundant cells in the RA synovium and the pannus of inflammatory vascular tissue. These immune cells are involved in both initiation and resolution of inflammation in RA, existing as the M1 and M2 phenotypes 2-5. *M1/M2* describes the two major and opposing activities of macrophages, namely, to kill/fight and to heal/fix. M1 phenotype macrophage is dominant over M2 in RA and produces the predominant pro-inflammatory cytokines (TNFα, IL-1β, and IL-6), together with chemoattractant factors (CCL2 and IL-8), matrix metalloproteinases (MMP-3 and MMP-12), transcription factors (NF-kB), and ROS. M1 macrophages promote Th1 cells, which in turn produce interferon (IFNγ) that strengthens and enhances M1 polarization in a feed-forward loop 3. Other contributor in secreting inflammatory cytokines and chemokines in RA synovium is monocytes migrating from blood to RA synovial tissues, where they differentiate into M1-macrophages and aggravate inflammation. Thus, repolarization of M1 macrophage and reprogramming of activated monocytes is a promising therapy. Macrophages possess the extraordinary plasticity and can change their phenotype in response to environmental stimuli or self-derived stimulating signals, with reversibly 2-5. Recent evidence suggests ROS plays its role in maintaining the homeostatic functions of macrophage and, in particular, macrophage polarization. Specifically, ROS promotes M1, and the defect of ROS production leads to M2 macrophage polarization followed by downregulation of TNFα and IL-1β in the surrounding environment 3, 4. Several researchers found that nanoceria treatment decreased TNFα, IL-1β, IL-1α, and iNOS expression in macrophages, increase arginase-1 (Arg-1) expression in macrophages, and shifted their activity towards M2-like polarization 17-19. It was reported that nanoceria scavenged intracellular ROS in PMA-activated monocytes as well 44. Other important factor for macrophage polarization is nanoceria's valence states ratio. Li et al revealed that an increase in the Ce^4+^/Ce^3+^ ratio elevates the polarization of the M2 phenotype of RAW264.7 macrophages, particularly for the healing-associated M2 percentage and anti-inflammatory cytokine secretion 18. The significant ROS scavenging potential (**Figure 5A**) of A-nanoceria and almost equal content of both valence states in its structure supported the speculation that our NPs could impart an immunomodulatory effect that allows regulating macrophage behavior and their polarization. Our speculation on A-nanoceria's abilities was verified on RAW 267.4 macrophage cells and THP-1 monocytes cells activated by PMA (**Figure 6**). Both tested cell lines treated with A-nanoceria indicated a shift to the M2-like phenotype (**Figure 6**) that was consistent with previous studies 17-19. Several general controls, where cells were treated with PBS, BSA and LPS/IFNγ for M1-like phenotype, and IL-4/IL-13 for M2-like phenotype (**Figure 6**, **Figure S3**), had been evaluated as well. Expression of two common markers, IL-1β and iNOS, associated with the inflammatory M1 macrophages, were reduced by A-nanoceria (**Figure S4**). There was an apparent switch in macrophage polarization towards the M2-like phenotype *in vivo*. Sections of inflamed tissues from the arthritic mice were analyzed by immunohistology displayed a high concentration of macrophages infiltrated into the paws (CD11b) (**Figure 7**). Expression of specific markers for M1 and M2 (iNOS and Arg-1 in **Figure 7A**) was compared between A-nanoceria treated arthritic mice and the PBS treated control. Arthritic mice treated with A-nanoceria contained a significantly lower number of M1 (iNOS) and a larger number of M2 macrophages (Arg-1) (**Figure 7B**) than the control group. These data correspond with qRT-PCR results (**Figure S4**). Thus, A-nanoceria was able to drive a favorable polarization of M0 macrophage to an M2-like phenotype that has high phagocytosis capacity, clear apoptotic cells, mitigate inflammatory response, and promote healing. It was found that ROS serves as a chemoattractant to monocytes and macrophage 51, and A-nanoceria, scavenging ROS, may reduce the number of migrating monocytes into RA sites. Moreover, downregulation on ROS by A-nanoceria may prevent monocytes differentiation into macrophage and ROS-dependent maturation of macrophages into the M1 phenotype.

### Animal imaging in the evaluation of A-nanoceria

**Figure 8:**
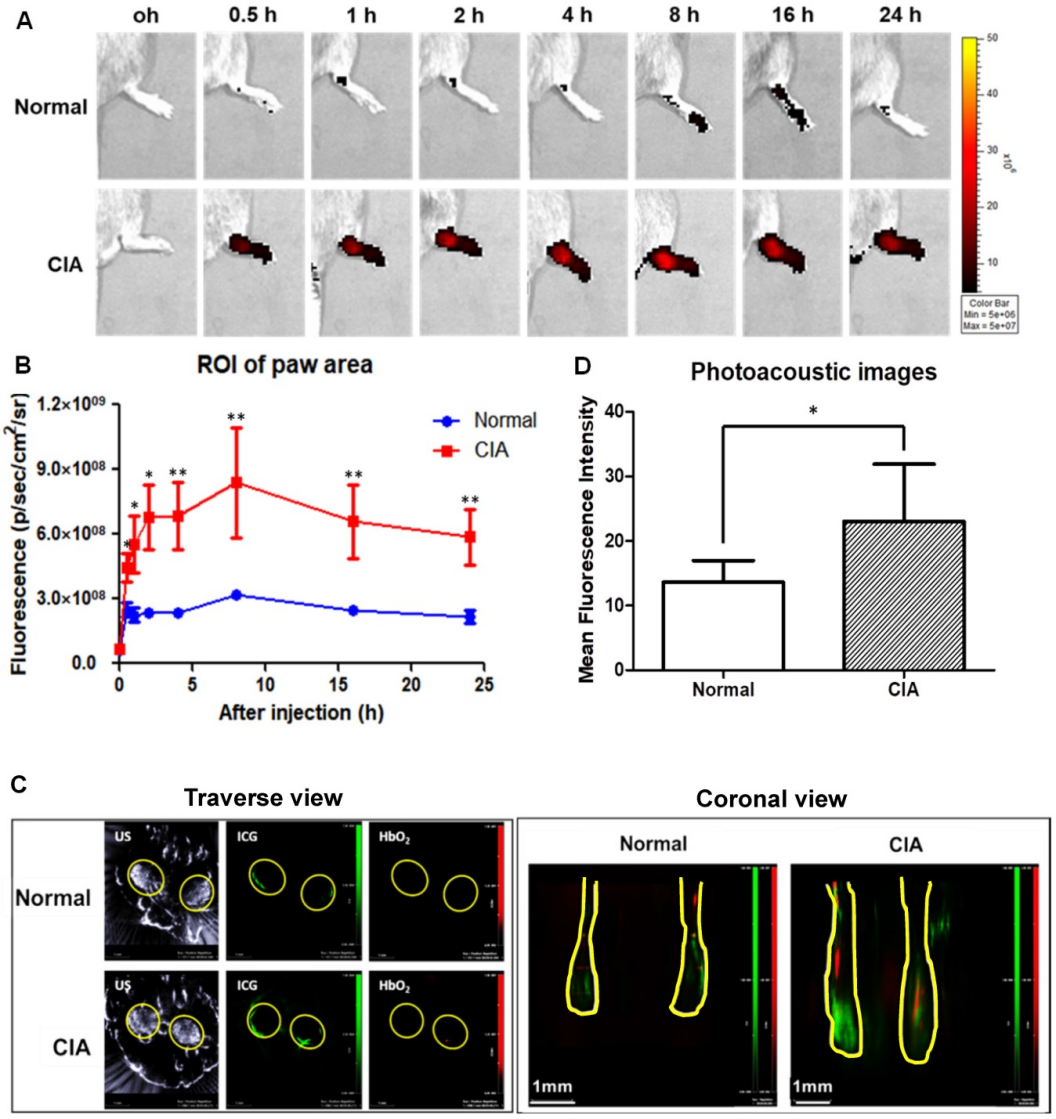
** Effect of targeted delivery of A-nanoceria in normal and CIA murine models (N = 3). (A)** IVIS images at different time point (0.5, 1, 2, 4, 8 , 16, 24 h) of ICG signal (red) after intravenous (IV) injection of A-nanoceria-ICG (50 µL) to the paws (yellow circle); **(B)** summarized plot of ICG signal in ROI (5 hind paws per a group) after IV injection of A-nanoceria-ICG into normal (blue trace line) and CIA (red trace line) mice built from (A); **(C)** MSOT traverse and coronal views of paws after IV injection of A-nanoceria-ICG (grey: ultrasound, green: ICG, red: oxygenated hemoglobin (HbO2)). Yellow circles and lines represent the paws of normal and CIA animals. **(D)** MFI signal of ICG plotted from 6 different spots from each image of MSOT images (p-value: *p < 0.05, **p < 0.005 compared with Normal). Both imaging modes, IVIS and MSOT, confirmed the high retention of A-nanoceria within inflamed zones.

Collagen-induced arthritis (CIA) murine model, a well-established animal model of human RA 52, was used for our *in vivo* studies (**Figure S5).** CIA animals were prepared using a previously published protocol 53, and their inflammatory profile was confirmed by immunohistology (**Figure 7** and **Figure S6**). We used this model to evaluate the accumulation of A-nanoceria in inflamed paws (**Figure 8**). The A-nanoceria-ICG exhibit dual-mode optical and optoacoustic signals from ICG fluorophore by IVIS and MSOT imaging system. Multi-spectroscopic images from MSOT can monitor the *in vivo* delivery and spatial distribution of injected nanoparticles 54. First, A-nanoceria-ICG behavior was explored in normal and CIA model mice. When intravenously injected, A-nanoceria displayed a higher accumulation in the paws of CIA mice compared to those of normal mice, confirmed by two imaging modalities (**Figure 8**). Normal mice showed lower ICG (A-nanoceria-ICG) than did CIA mice until 24 h. The uptake of A-nanoceria-ICG was increased at early time points (8 h), and then slowly decreased in paws of both normal and CIA mice (**Figure 8A-B**). The overall half-life of A-nanoceria-ICG had a similar tendency, a decrease by 24 h (**Figure 8A-B**), for both models. MSOT images of the oxygenated hemoglobin content, an indicator of the blood flow 55, revealed that CIA mice expressed higher ICG signals than normal mice (**Figure 8C-D**). Both oxygenated hemoglobin and ICG were located in similar regions presented in the transverse view of images. These results suggest that CIA mice had more blood flow in paws than normal mice 56, 57, and nanoceria particles accumulated in inflammatory regions through the blood vessels (**Figure 8C-D**). Similarly, in a previous study, J. Vonnemann *et al.* demonstrated the photoacoustic signals from the injected gold nanorods are trackable in accumulation to RA sites aligned with the oxygenated hemoglobin signals from MSOT images 58, 59. Taken together, these data suggest that there are effective delivery and accumulation of A-nanoceria to the inflamed and swollen sites of CIA animals as a model of human RA.

### Inflammation-targeting feature of A-nanoceria-ICG

**Figure 9:**
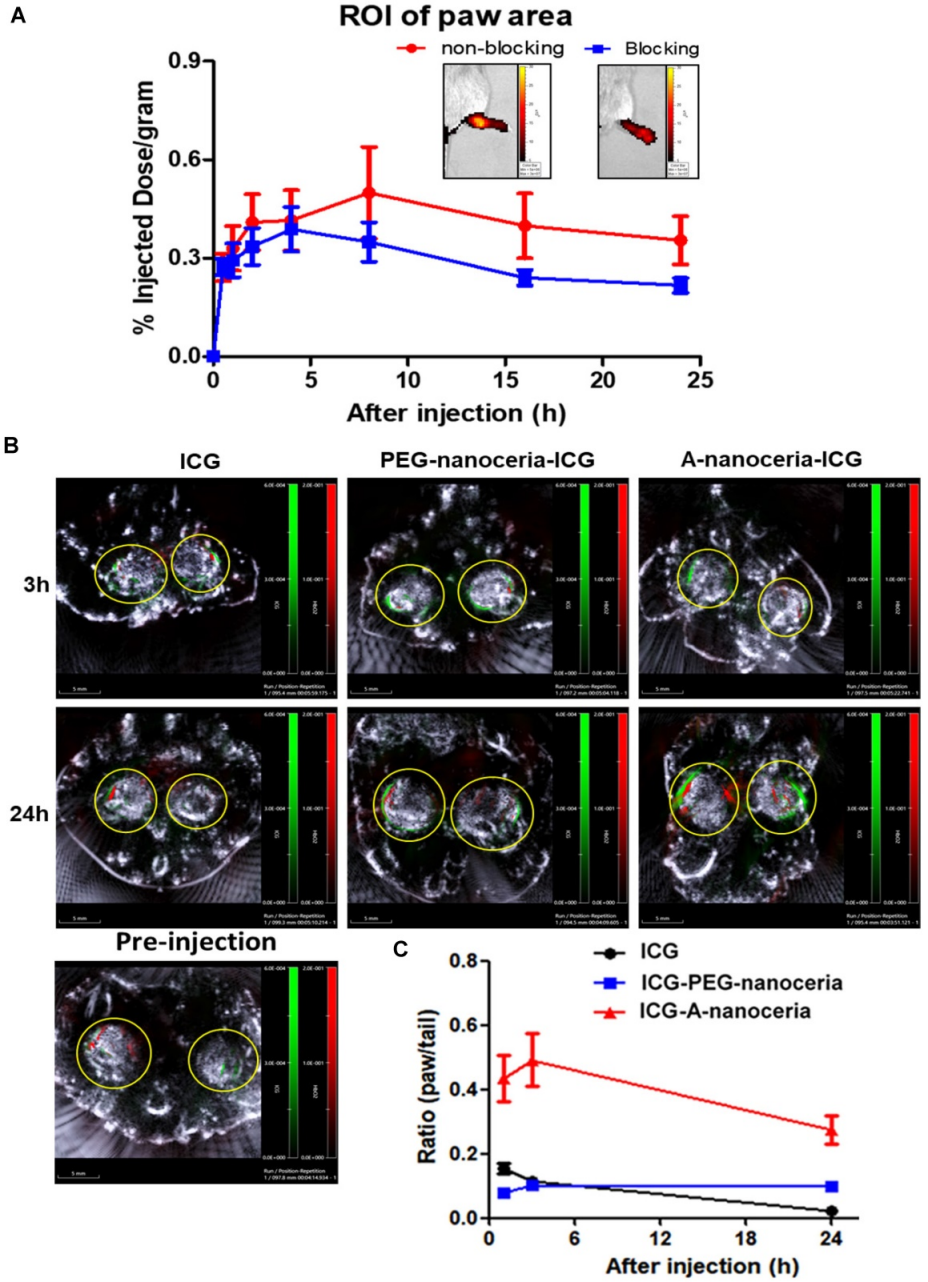
** Evaluation of targeting efficiency of A-nanoceria. (A)** Confirmation of SPARC-associated mechanism of A-nanoceria uptake and its accumulation within inflamed zones using IVIS. A-nanoceria uptake was inhibited by blocking SPARC using anti-SPARC antibody (50 µg by IP injection with precursor A-nanoceria); the insets: IVIS images of the hind paws of CIA animals treated with A-nanoceria (non-blocking) and anti-SPARC+A-nanoceria (blocking). (N = 3 mice per group, 5 hind paws per each; error bars = SEM). **(B)** Distribution of ICG, A-nanoceria-ICG and PEG-nanoceria-ICG over time by MSOT. The images (scale bar is 5 mm) represent the overlay of three signals (grey: ultrasound, red: HbO_2_, green: ICG) in the animal paws before and after (3, 24 h) tail vein injection of ICG (50 µL), A-nanoceria-ICG (50 µL), or PEG-nanoceria-ICG (50 µL) at equal amount of ICG injected per a treatment (concentrations and injection volume). Yellow circles represent the cross-section of the hind paws. Intensive ICG signal can be notified around HbO_2_ signal in the A-nanoceria-ICG treated animal paw. **(C)** Summarized plot of ICG signal distribution in the ROI of the paws and the tail (ratio between paw and tail) from IVIS imaging data (**Figure S7**). ICG signal distribution in the paws and the tail from ICG-PEG-nanoceria is included as a control group (N = 3, error bars = SEM).

Second, the inflammation target efficiency of A-nanoceria was assessed in the CIA model. ICG-labeled A-nanoceria accumulated well in swollen and inflamed joints of CIA animals (**Figure 8**) due to albumin substrate that can increase the blood circulation and retention of NPs into inflamed zones. In the pathology of RA, various types of cells, including inflammatory cells (e.g., macrophages and T cells) and vascular endothelial cells, are activated and with an associated change expression of cell surface receptors, including those for scavenging albumin (gp18, gp30, SPARC, FcRn, gp60, the Megalin/Cubilin complex) 25-26—these receptors increase endocytosis, transcytosis, and catabolism of albumin. Elevated gp60 and SPARC in the synovium fluid of RA patients and murine CIA model work to transports albumin across the endothelial barrier into the interstitium (Gp60) and SPARC enhances the uptake of albumin into immune cells in the RA synovium 24, 26. Albumin-based NPs can be taken up *via* the SPARC-mediated mechanism 60 and directly internalized by phagocytic cells *via* interactions with FcRn receptors, or after opsonization by IgG, and eventually sequestered within the endo-lysosomal compartment. The overall scheme of the NPs interactions and internalization within the RA site is presented in **Figure 1C**. To confirm the SPARC-associated mechanism of A-nanoceria uptake and targeting inflamed zones, we blocked SPARC in CIA animals by anti-SPARC antibody (50 µg by IP injection with precursor A-nanoceria; blocking on **Figure 9A**) and compared these animals with the control group treated with only ICG-conjugated A-nanoceria (non-blocking on **Figure 9A**). After blocking, there was a decrease of ICG signal from 4 h later, although it was not statistically validated. To elucidate further albumin mediated distribution of A-nanoceria specifically into RA joints, we performed a comparative study on the distribution of free ICG, A-nanoceria-ICG, and PEG-nanoceria-ICG in CIA mice using IVIS and MSOT (**Figure 9B-C**). A-nanoceria-ICG accumulated to higher levels within inflamed paws than the other two formulations (**Figure 9** and**Figure S7**). Albumin of A-nanoceria-ICG correlated with more accumulation and retention of NPs in the swollen paws of CIA mice. The 3 h post-injection, A-nanoceria-ICG was slowly eliminated from the inflamed regions, as evidenced by both imaging modalities (**Figure 9** and **Figure S7**). ICG signals from A-nanoceria-ICG are greater than those for ICG alone and PEG-nanoceria-ICG (**Figure 9**). A-nanoceria-ICG demonstrated a long-life circulation in the blood and extended presence in the paws due to albumin substrate; ICG signals were still apparent after 24h (**Figure 9**). Free ICG dye did not accumulate within RA regions, and localized signals were dramatically lower at the 3 h time point and near 0 by 24 h post-injection (**Figure 9**). PEG-nanoceria-ICG also accumulated in the paws and was slowly eliminated from the inflamed regions (**Figure 9C** and**Figure S7**); this was confirmed with MSOT imaging as well (**Figure 9B**)**.** The optical signals from PEG-nanoceria-ICG exceeded that of A-nanoceria-ICG (**Figure S7**) but localized most to the tail and did not accumulate well in the inflamed paws. The albumin substrate altered the bio-distribution of the NPs, directing A-nanoceria delivery and accumulation to afflicted joints likely *via* elevated albumin and scavenging receptors (**Figure 1C**) with increased duration of retention for NPs in inflamed zones (**Figure 8** and** 9**) for at least 24 h. The RA sites have loose vasculature and increased demand in nutrients, such as albumin, those likely accounts for our observations and generally impact nanomedicine approaches in treating inflammatory diseases. Thus, our A-nanoceria-ICG could be a generalizable inflammation-targeting contrast and therapeutic agent.

### Therapeutic efficacy of A-nanoceria in CIA model

**Figure 10:**
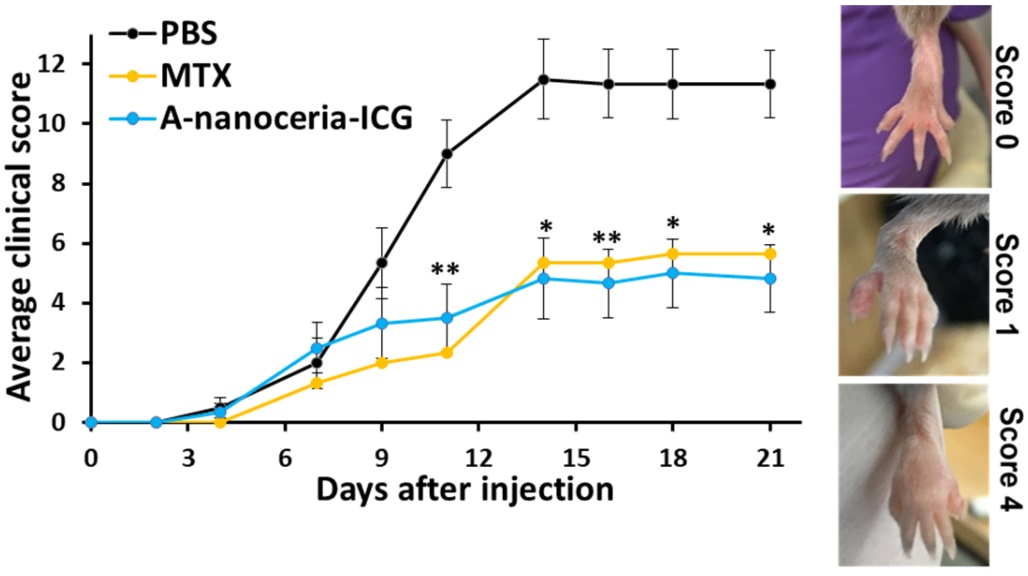
** Therapeutic effect of A-nanoceria and its comparison with MTX and PBS treated groups of CIA mice based on clinical scores evaluation (N = 6).** After three weeks, A-nanoceria-ICG treated group (50 µL, blue line) showed 4.8 ± 1.1 average clinical score, similar to MTX group (50µL, yellow line), that is 2.4-fold lower than PBS control group (black line). The inset shows the representative images of CIA mice to assess the clinical scoring of paw swelling with error bars as SEM (*p*-value: *p < 0.05, **p < 0.005 compared with PBS).

Any therapeutic effects of A-nanoceria are directly associated with ROS scavenging (**Figure 5A**), oxidant/antioxidant balance *via* its enzymatic activity (**Figure 5B**), and polarization of macrophages to normalize the M1/M2 ratio (**Figure 7**, **Figure S4** and** S6**) to resolve inflammation of the joints. The potential therapeutic effects of A-nanoceria was compared to that of the drug methotrexate (MTX) 6, 19, 61 taken in the same concentrations, and to PBS treated groups of CIA mice (**Figure S5**). The MTX dose was adopted from the previous studies utilized the same RA model and revealed the efficacy in that particular MTX dose for such a model 62, 63. A-nanoceria dose was taken in an equal amount to MTX dose to compare the effectiveness of both therapy modes. The CIA model has been adapted to assessing efficacy using a clinical scoring scheme similar to that used for the evaluation of RA symptoms and disease development 53. The clinical score scale ranged from 0 (initial stage) to 4 (severe stage), and an example for RA severity evaluation is given in **Figure 10 (inset)**. Over time, the clinical score sharply increased in the saline group, and a mild increase was observed in the A-nanoceria and MTX treated groups (**Figure 10**). The A-nanoceria treated group showed a score similar to the one observed for the saline treated group over the first 6 days and then dropped well below at 7 day post-treatment. The A-nanoceria treated group had scores slightly higher than those observed for the MTX treated groups until day 12. After day 12, the A-nanoceria treated group had scores similar to the MTX treated group. A-nanoceria and MTX treated groups demonstrated a reduction in symptoms by dropping clinical scores of arthritis in contrast to the saline treated group (**Figure 10**). The average clinical score of the nanoceria group (4.8±1.1) and MTX group (5.6±4.2) was significantly lower rather than the saline group (11.3±1.5) at 21 days after treatment (**Figure 10**). An additional control study on changes in clinical scoring was performed for CIA animals treated with albumin (**Figure S8**), and no beneficial effect (p = 0.8335) was observed for that treatment. Thus, MTX and A-nanoceria treatments have similar therapeutic efficacy; however, A-nanoceria-ICG benefits from its targeting and ability to be imaged. Unlike A-nanoceria, MTX does not accumulate in RA-associated zones. Generally, MTX has a short plasma half-life (1.5-3.5 h) and accumulates in normal tissues, especially liver, kidneys, and intestine producing severe toxicities like ulcerative colitis, hepatotoxicity, and nephrotoxicity 64, 65. In addition to these non-selective toxicities, patients treated with MTX are prone to resistance, limiting its effectiveness. It has been reported that 30% of the treatments' remission failures are due to MTX resistance 66.

Hypoxia is another RA feature and is initiated when tissue expansion and cellular metabolism increase the oxygen demand exceeding supply. Hypoxia induces profound changes in gene expression in RA; in particular, HIF-1α and VEGF are increased. Activation of HIF signaling leads to changes in the expression of about 1% of all human genes. The genes affected by HIF include those involved in energy metabolism, pH regulation, erythropoiesis, angiogenesis, and apoptosis, which together allow cells, tissues, and organisms to adapt to reduced oxygenation 4. The increase in RA synovial tissue mass, due to a combination of synovial fibroblasts hyper-proliferation (VEGF) and infiltration by cells derived from the circulation, including T cells and myeloid cells, lead to elevated oxygen consumption in the RA synovium. Our results confirmed that the treatment of pro-inflammatory M1 macrophages with A-nanoceria dramatically decreased the HIF-1α protein content (**Figure S9**). The synovial fluids from patients with RA were reported to be characterized by low oxygen tension (2-4% compared to 9-12% in patients without RA) 2-4. Recently J. Kim confirmed that nanoceria could induce oxygenation *via* conversion of hydroxyl radicals to O_2_ during the Fenton reaction in a hypoxic environment 12. In this study, we evaluated oxygenation by MSOT and observed improvements in the paws of A-nanoceria treated animals (**Figure 8C** and** 9B**). Therefore, the therapeutic effects of A-nanoceria may be *via* oxygen delivery to 'starving' RA tissues. Hyperbaric oxygen therapy has been demonstrated to improve RA 67, and A-nanoceria would be a complex alternative treatment of RA. The combination of O_2_ generation and ROS scavenging may enable A-nanoceria to restore the oxygenation of diseased synovium to normal levels and thus further modulate the hypoxia-dependent pathologies of RA.

## Conclusions

This study describes the development of novel ceria based theranostic nanoparticles for rheumatoid arthritis prevention and treatment. The preparation of these nanotheranostics is straightforward and with simple chemistry. The nanotheranostics possess high colloidal stability, reproducibility and can be easily scaled up. The uniqueness of the nanotheranostic is its antioxidant and enzymatic properties owing to the formation of a new enzymatic structure by albumin and nanoceria. The A-nanoceria could be delivered systemically with accumulation in synovial tissues of joints through the SPARC-mediated mechanism, and effectively inhibit inflammation *via* reducing hypoxia, scavenge excessive ROS, and restoring the misbalance of M1/M2 macrophages. Moreover, the nanoparticles are non-invasively trackable for evaluating the therapeutic delivery and bio-distribution by optical/optoacoustic imaging. Taken together, these multifunctional nanotheranostics may constitute an effective alternative to the current DMARDs in RA therapy.

## Supplementary Material

Supplementary figures and tables.

## Abbreviations

RA: rheumatoid arthritis
nanoceria: cerium oxide nanoparticles
BSA: bovine serum albumin
A-nanoceria: BSA-cerium oxide nanoparticles
ICG: indocyanine green
IVIS: *in vivo* imaging system
MSOT: multi-spectral optoacoustic tomography
ROS: reactive oxygen species
gp60: glycoprotein 60
SPARC: secreted protein acidic and rich in cysteine
NPs: nanoparticles
VEGF: vascular endothelial growth factor
NF-kB: nuclear factor KappaB
RT-PCR: reverse transcription polymerase chain reaction
IL: interleukin
MMPs: matrix metalloproteases
TNF: tumor necrosis factor
NSAIDs: non-steroidal anti-inflammatory drugs
DMARDs: disease-modifying antirheumatic drugs
MTX: methotrexate
SOD: superoxide dismutase
iNOS: inducible nitric oxide synthase
LPS: lipopolysaccharides
IFNγ: interferon-gamma
PMA: phorbol 12-myristate 13-acetate
PBS: phosphate-buffered saline
