# Supplementary material for: Ceria-based nanotheranostic agent for rheumatoid arthritis
Source: Theranostics. 2020 Oct 25;10(26):11863–80. doi: 10.7150/thno.49069 (PMC7667692; doi:10.7150/thno.49069)
Supplement: Supplementary file 1 — Supplementary figures and tables. [file thnov10p11863s1.pdf]

Supplementary Materials for:

Ceria-based nanotheranostic agent for rheumatoid arthritis

Irina Kalashnikova[1†], Seock-Jin Chung[1†], Md Nafiujjaman[1], Meghan L. Hill[1], Mzingaye E. Siziba[1], Christopher H. Contag[1,2], and Taeho Kim[1*]

[1]*Department of Biomedical Engineering and the Institute for Quantitative Health Science & Engineering,* [2]*Department of Microbiology and Molecular Genetics, Michigan State University, East Lansing, MI 48824, USA*

**Corresponding Author: kimtae47@msu.edu*

[†]*I.K. and S-J.C. contributed equally to this work.*

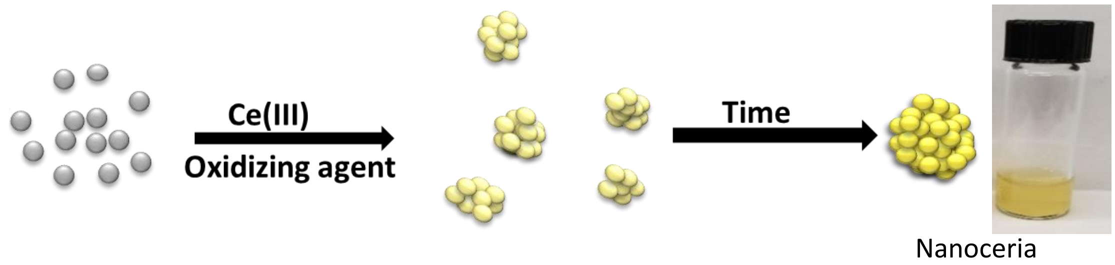

Figure S1. Schematic representation for nanoceria synthesis without albumin substrate presence. The inset shows a picture of nanoparticle (nanoceria) suspension from this synthesis, which is turbid due to the low solubility.

Signal	Signal identity, relevant samples
**IR data (cm^{-1}; s = shift; ν = stretching; δ = bending)**	
3290 s 3281	ν (N-H)
1700–1600	ν (C=O)
1522 s 1537	ν (C–N), δ(C-NH)
1642 s 1645	α-helix of BSA
1645, 1362	A-Ce^{3+} complex
1348 s 1318	Ce=O, ν(C–N), δ(C-NH)
1039 to 1041	Ce=O
928	N (C-C) proline, valine
851 s 847, 843 s 838,	δ,ν (Ce-O)
825	ν (Ce-O-C)
729 s 730	ν (Ce–O–Ce)
634 s 627, 619 s 618	δ (Ce–O–C)
581 s 584, 548 s 551, 524 s 523	ν (Ce-O)
**UV-Vis (a.u.; s = shift)**	
232, 278	Albumin
249 s 253	Ce^{3+}
273 s 271	Ce^{3+}
305 s 307	Ce^{4+}
526	Doping by ceria
667 s 728	ICG

Table S1. Summarized data on spectroscopies analysis for FT-IR (top) of A-nanoceria and for UV-Vis (bottom) of A-nanoceria-ICG (associated with **Figure 3A-B**).

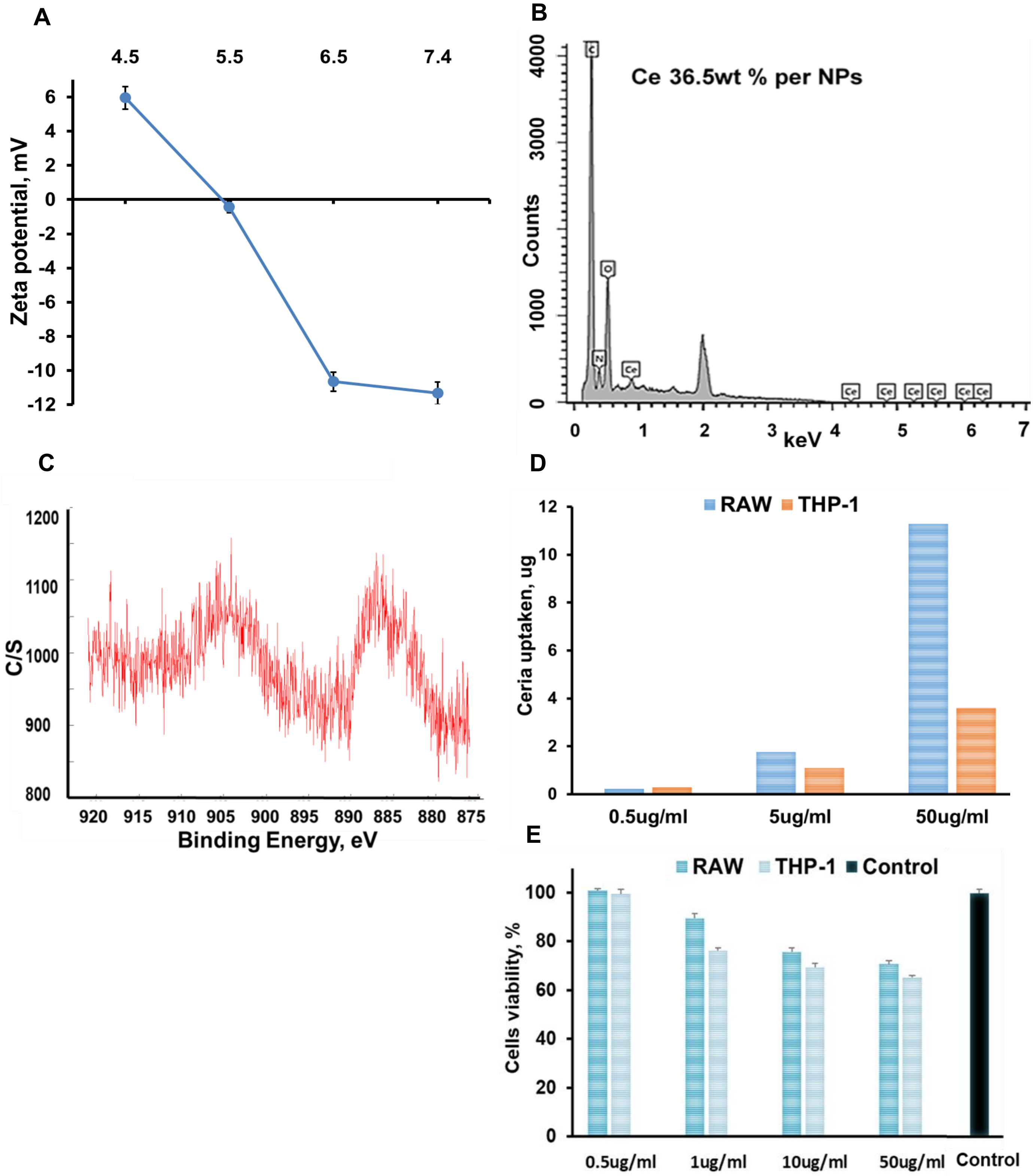

Figure S2. Additional characterization of **(A)** Zeta potential of A-nanoceria at different pH (4.5, 5.5, 6.5, and 7.4); **(B)** EDS analysis determined about 36.5% of Ce element per NPs (A-nanoceria); **(C)** Full XPS spectra of A-nanoceria (related to fitted data presented on **Figure 4C**); **(D)** NPs uptake measured by Ce signal was detected by ICP-OES. RAW 264.7 and THP-1 cells were treated with different concentrations of A-nanoceria (0.5, 5, and 50 ug/mL) and the results showed a concentration-dependent uptake, more intensive in the case of RAW cells;

(E) MTT cytotoxicity assay (black: untreated cells, blue: RAW 267.4 cells and light blue: THP-1 cells treated with 0.5, 1, 10, 50 µg/mL A-nanoceria, where N = 10, error bars = SD The 1.4 and 1.6-fold decrease in RAW and THP-1 cells, respectively, was revealed after treatment with 50 µg/mL A-nanoceria.

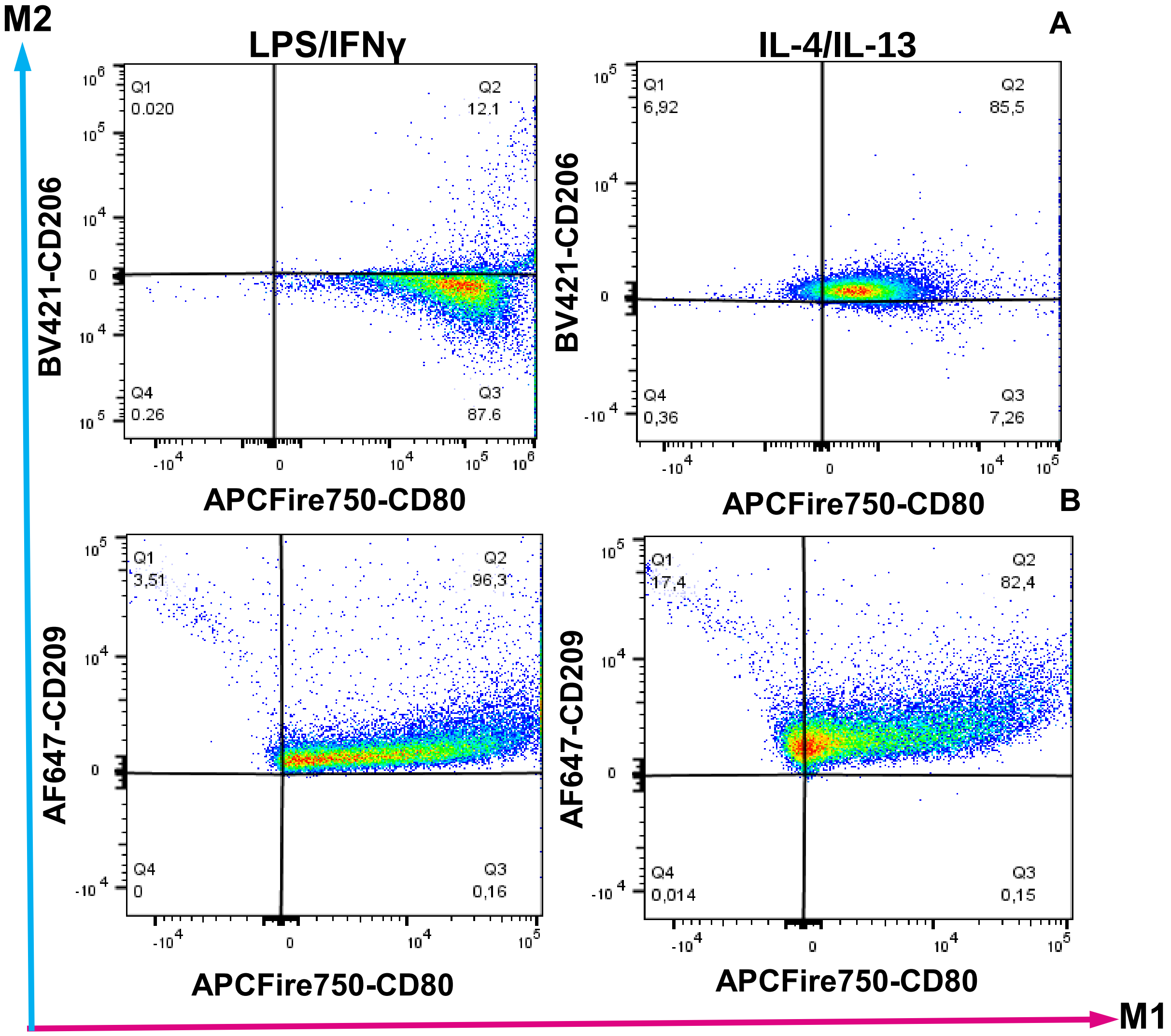

Figure S3. Flow cytometry analysis of **(A)** RAW 264.7 cells and **(B)** THP-1 cells: cells were treated with LPS/IFN-γ and IL-4/IL-13 for 24 h moving cells to M1- and M2-like phenotypes, respectively (see Q2/Q3 and Q1).

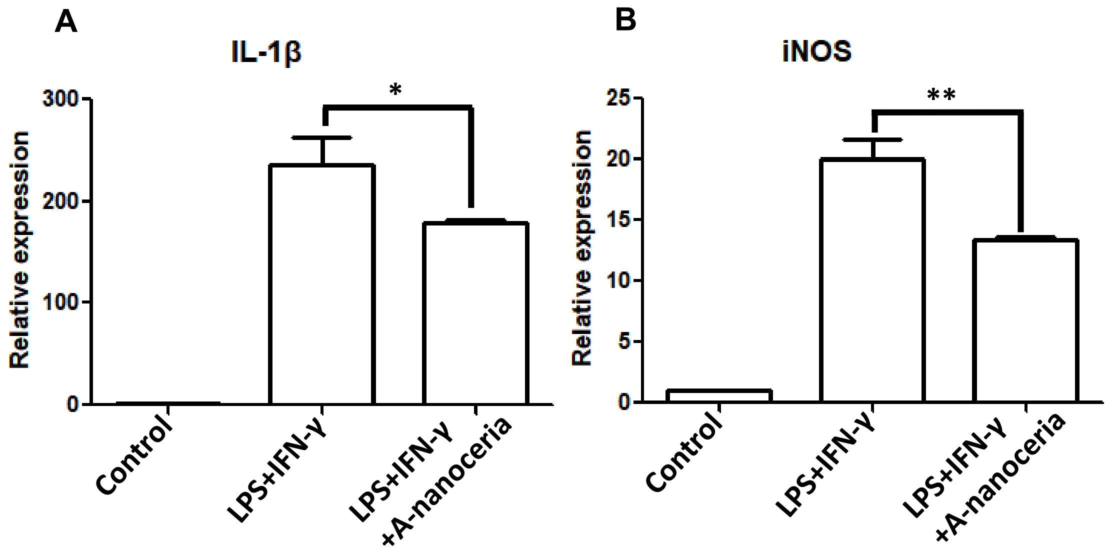

Figure S4. qRT-PCR analysis of **(A)** IL-1β and **(B)** iNOS expression in RAW 264.7 cells: untreated, treated with LPS/IFN-γ and LPS/IFN-γ/A-nanoceria for 24 h. Both M1-phenotype (activated by LPS/IFN-γ) markers, IL-1β and iNOS, were decreased by A-nanoceria treatment (N = 3, error bar = SD, *p < 0.05, **p < 0.005).

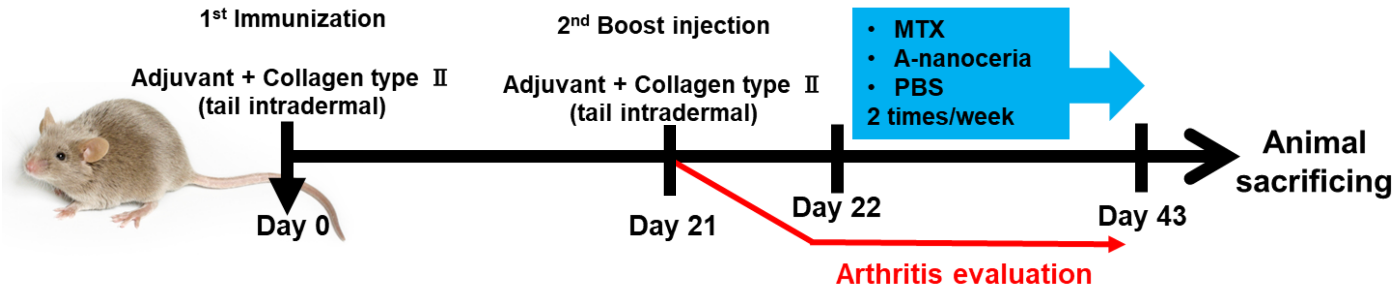

Figure S5. Animal study: full time range including CIA model preparation and further study with RA clinical scoring evaluation. The adjuvant and collagen II were injected intra-dermally twice at day 0 and day 21, after that animals developed RA signs and study started with intra-articular injection of A-nanoceria or PBS. MTX solution was injected intraperitoneally. Animal were measured their clinical scores three time a week from 21 day to 43 day of the study.

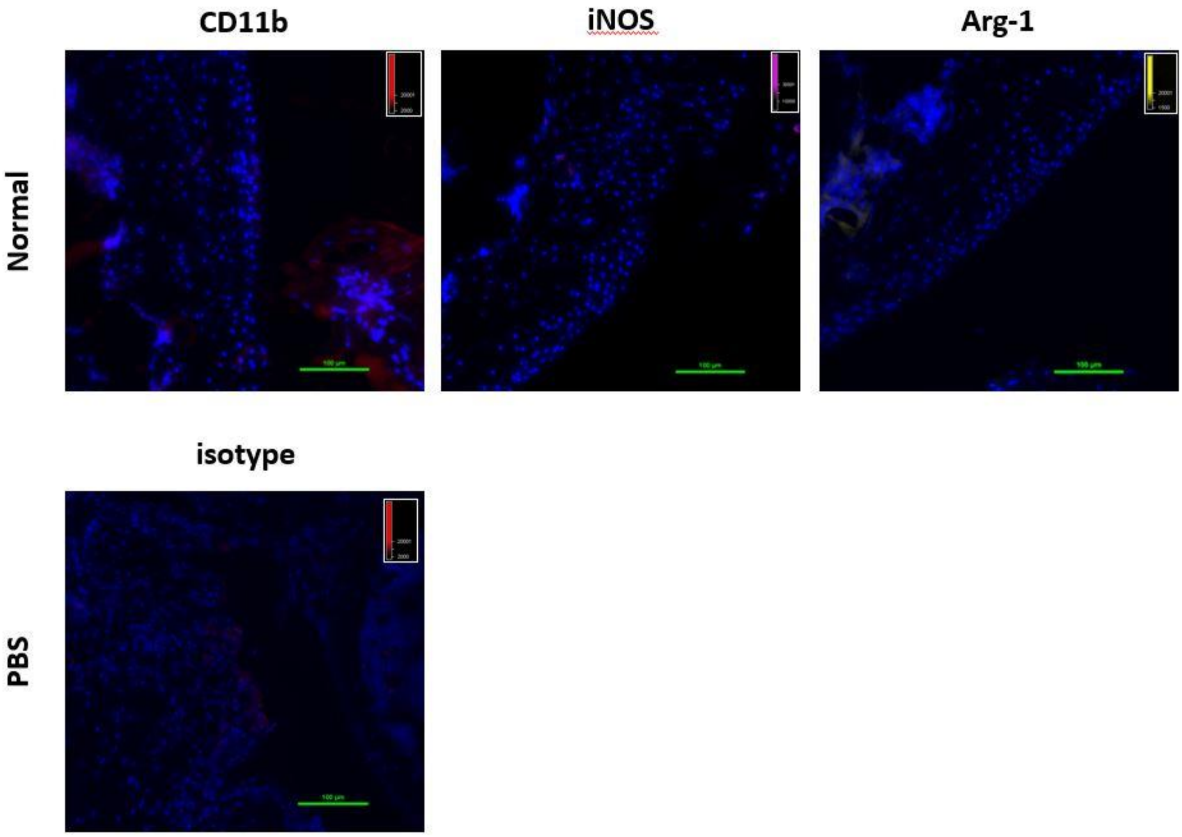

Figure S6. Immunohistology analysis of tissue sections harvested from normal mice: with no treatment or after treatment with PBS. DAPI is in blue, CD11b and isotype are in red, iNOS is in pink, and Arg-1 is in yellow. Scale bar = 100μm.

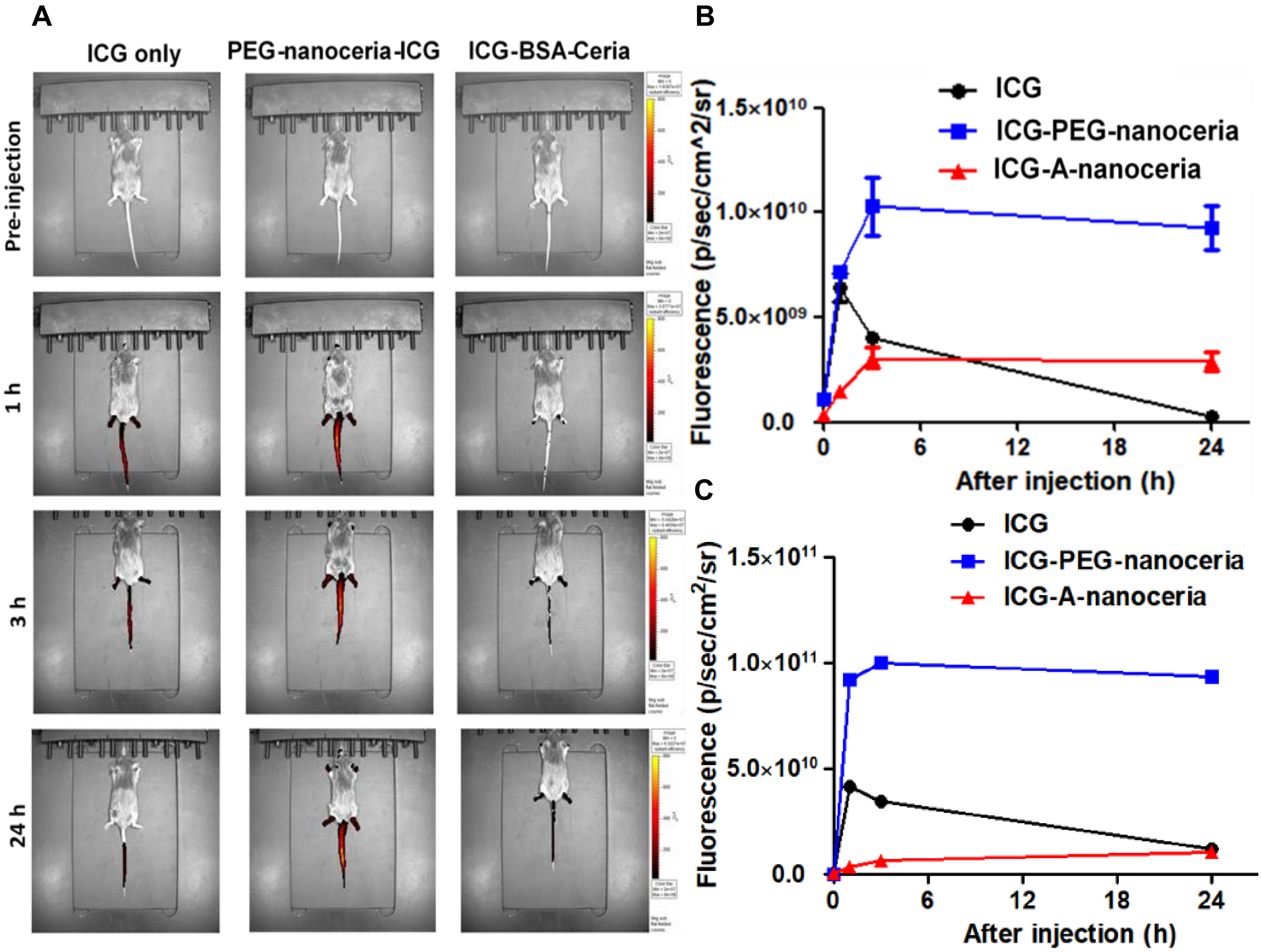

Figure S7. IVIS analysis of ICG signal for three formulations: ICG; ICG-PEG-nanoceria; ICG-A-nanoceria. **(A)** images of ICG signal in CIA animals after tail vein injection at 1, 3, and 24 h time points; **(B)** the plots of raw ICG signal intensity in the arthritic paws of CIA mice by three formulations from **(A)** where N = 3, error bars = SEM; **(C)** plot of raw ICG signal intensity in the tail of CIA mice by three formulations from **(A)**. Black: ICG; blue: ICG-PEG-nanoceria; Red: ICG-A-nanoceria.

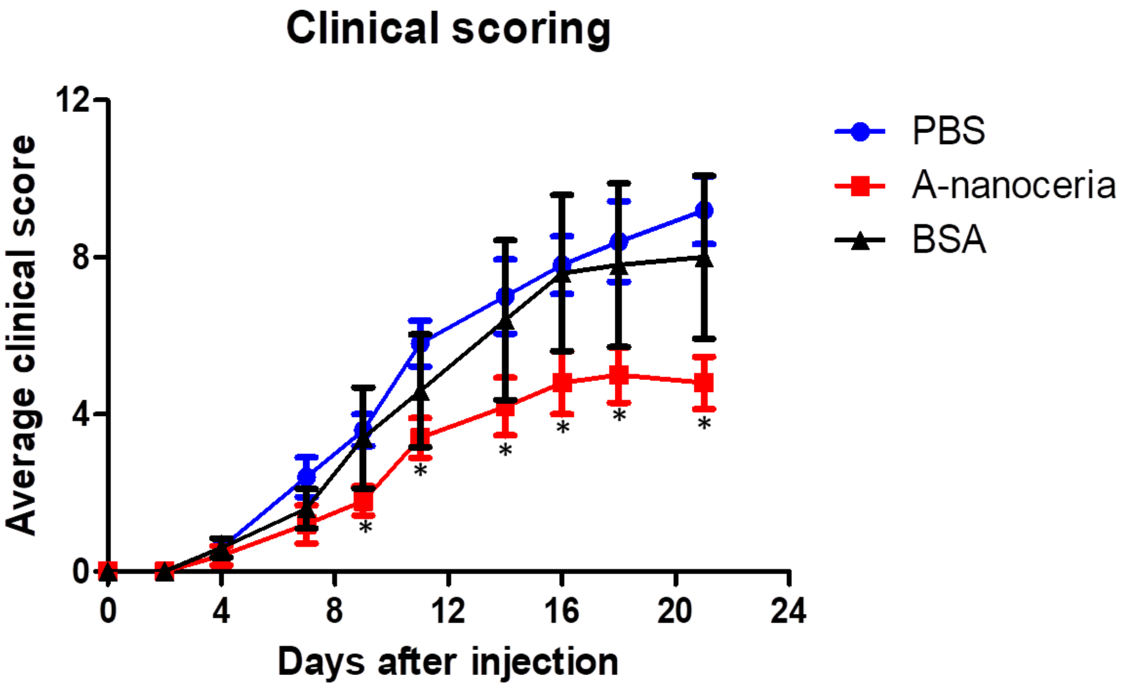

Figure S8. Clinical scoring alteration over 21 days course of CIA mice treatment with A-nanoceria and two controls (BSA and PBS). (N = 5 mice per group). After three weeks of the treatment course, A-nanoceria-ICG treated group (50 µL, red line) showed significantly low clinical score than PBS and BSA control groups (50 µL, blue and black lines, respectively). Albumin itself has no valuable effect on RA recovery (Error bars = SEM and p-value from Mann-Whitney U test: * $p < 0.05$ compared with PBS).

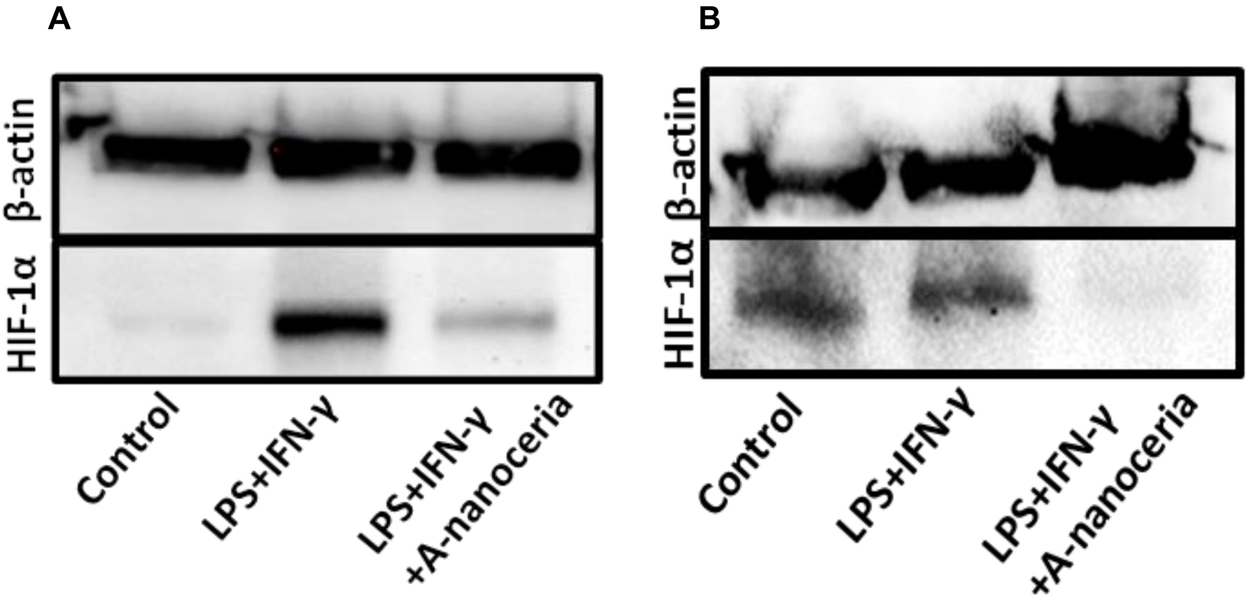

Figure S9. Western blot of HIF-1α protein expression in **(A)** RAW 264.7 and **(B)** THP-1 cells: untreated, treated with LPS/IFN-γ, and treated with LPS/IFN-γ/A-nanoceria for 24 h. All samples were normalized to β-actin expression as an internal reference. Treatment of activated macrophage with A-nanoceria demonstrated downregulation of HIF-1α expression level.
